# Global trends of antimicrobial resistance rates in *Neisseria gonorrhoeae*: a systematic review and meta-analysis

**DOI:** 10.3389/fphar.2024.1284665

**Published:** 2024-07-03

**Authors:** Mohammad Hosseini Hooshiar, Mohammad Sholeh, Masoumeh Beig, Khalil Azizian, Ebrahim Kouhsari

**Affiliations:** ^1^ Department of Periodontology, School of Dentistry, Tehran University of Medical Sciences, Tehran, Iran; ^2^ Department of Bacteriology, Pasteur Institute of Iran, Tehran, Iran; ^3^ Department of Microbiology, Faculty of Medicine, Kurdistan University of Medical Sciences, Sanandaj, Iran; ^4^ Zoonoses Research Center, Research Institute for Health Development, Kurdistan University of Medical Sciences, Sanandaj, Iran; ^5^ Laboratory Sciences Research Center, Golestan University of Medical Sciences, Gorgan, Iran; ^6^ Department of Laboratory Sciences, Faculty of Paramedicine, Golestan University of Medical Sciences, Gorgan, Iran

**Keywords:** *Neisseria gonorrhoeae*, antimicrobial resistance, systematic review and meta-analysis, spectinomycin, gonorrhea

## Abstract

**Background:**

Antimicrobial resistance (AMR) of *Neisseria gonorrhoeae* (NG) is a significant public health concern.

**Objective:**

The objective of our study was to assess global AMR rates and test them both temporally and geographically.

**Methods:**

We conducted a systematic search of relevant reports from international databases up to 2021. The R statistical package was used for all statistical analyses.

**Results:**

A total of 225 articles were analyzed, and 432,880 NG isolates were examined. The weighted pooled resistance (WPR) rate of different antibiotics was as follows: ciprofloxacin, 51.6%; tetracycline, 45.4%; trimethoprim/sulfamethoxazole, 42.4%; chloramphenicol, 4.1%; kanamycin, 2.1%; gentamicin, 0.6%; and spectinomycin, 0.3%. The resistance to spectinomycin, gentamicin, and kanamycin decreased over time. Significant differences in antibiotic resistance rates were found between the countries.

**Conclusion:**

Our findings reveal a continuous increase in resistance to some antibiotics (tetracycline and ciprofloxacin) historically used for gonorrhea, even after discontinuation. However, encouraging trends of decreasing resistance to spectinomycin, gentamicin, and kanamycin were observed. Continued global monitoring of AMR profiles in NG isolates is essential for informing appropriate treatment strategies and mitigating the threat of untreatable gonorrhea.

## Introduction

Gonorrhea, the second most common bacterial sexually transmitted infection (STI), is a major public health challenge (de Munain, 2019). According to the World Health Organization (WHO), there were over 80 million new cases of gonorrhea worldwide by 2020 (Whelan et al., 2021). The WHO Global Gonococcal Antimicrobial Surveillance Programme (GASP) has monitored the global increase and development of antimicrobial resistance (AMR) in gonorrhea since 1992. Its aim is to assess AMR status, identify emerging AMR, and make changes to clinical guidelines and public health strategies at the national and international levels (Organization, 1990). The WHO Gonococcal AMR Surveillance Programme (WHO-GASP) has been supported and expanded to all WHO regions since 2009 (Unemo et al., 2019a). In many countries, there is an increasing emergence of reduced susceptibility or resistance to antibiotics, which are currently recommended for treatment (Workowski et al., 2008; Unemo, 2015). Various therapies, such as ceftriaxone, azithromycin, gentamicin, kanamycin, tetracycline, chloramphenicol, spectinomycin, ciprofloxacin, and trimethoprim/sulfamethoxazole, are recommended at different times for the treatment of gonorrhea (Unemo, 2015; Fifer et al., 2016; Unemo et al., 2019b). However, resistance to available treatment regimens is increasing, making successful treatment difficult (Hsu et al., 2022; Lu et al., 2022). The prevalence and rapid growth of AMR in *Neisseria gonorrhoeae* (NG) have been widely reported worldwide. This has led to limited treatment options for empirical therapy, resulting in an increase in severe complications such as infertility, ectopic pregnancy, and the spread of the human immunodeficiency virus (HIV) (Unemo and Shafer, 2014a; Unemo et al., 2016; Ngobese and Abbai, 2021). Our meta-analysis investigated global trends in AMR for NG, focusing on seven antibiotics: ciprofloxacin, spectinomycin, trimethoprim/sulfamethoxazole, tetracycline, gentamicin, kanamycin, and chloramphenicol. Ceftriaxone and azithromycin, the current first-line treatments for gonorrhea, were excluded due to emerging global resistance and the need for alternative treatment options.

Our study evaluated the efficacy of these seven antibiotics as potential alternatives in cases of ceftriaxone and azithromycin resistance. Despite current guidelines recommending ceftriaxone and azithromycin, resistance is increasing in some regions, necessitating alternative therapies.

We acknowledge the importance of ceftriaxone and azithromycin in treating gonorrhea and plan to include them in the future when more data on their efficacy and resistance patterns are available. Our primary objective was to assess global antimicrobial resistance trends in NG and explore alternative treatments in response to emerging resistance. We aim for our study to contribute to efforts to monitor and address AMR in gonorrhea treatment.

Estimating global resistance rates for NG is vital to the development of active, accessible, and affordable treatments by

1. Identifying regional resistance patterns to guide local treatment guidelines.
2. Prioritizing research and development of new antibiotics and alternative therapies.
3. Monitoring the effectiveness of existing treatments and revising guidelines when necessary.
4. Raising awareness and promoting responsible antibiotic use to slow down resistance development.

These efforts ultimately contribute to improved patient outcomes and reduced healthcare burden.

## Methods

This systematic review adheres to the guidelines of the Preferred Reporting Items for Systematic Reviews and Meta-Analyses (PRISMA) (Moher et al., 2009).

### Search strategy and study selection

**FIGURE 1:**
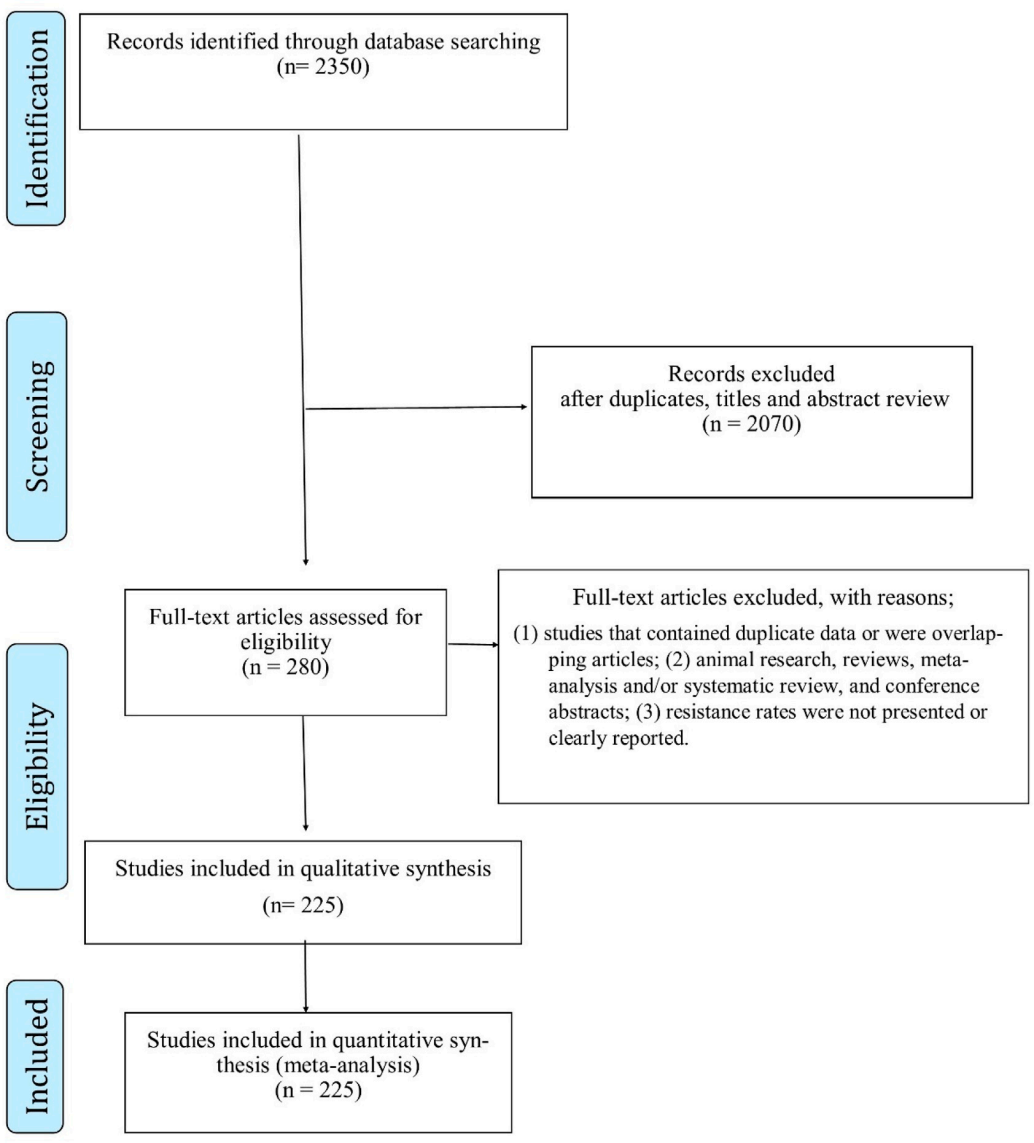
Flowchart of study selection.

Electronic databases, such as MEDLINE, Scopus, and Web of Science, were used for this study. The search was performed using the terms “*N. gonorrhoeae*,” “gonorrhea,” or “Gonococcus” in combination with “antibiotic resistance” in the title, abstract, and keyword fields. The search included articles published between 1988 and 2021. Boolean operators were used to combine the descriptors. The search strategy was customized to match the specific characteristics of each database. Synonyms were searched before each keyword, and a search option was used to identify similar terms. There were no limitations to the database search. The records obtained from the database search were merged, and duplicate entries were removed using EndNote X9 (Thomson Reuters, New York, NY, United States). Furthermore, the reference lists of the eligible articles were reviewed to identify potentially relevant studies. The authors also checked the reference lists of articles to ensure that no additional studies were overlooked in the initial search. One reviewer conducted the searches, and two independent reviewers performed an initial screening of potentially relevant records based on the titles and abstracts using the inclusion/exclusion criteria. Full articles were extracted from the datasets and screened for relevance by two independent reviewers. Disagreements with a third reviewer were resolved by consultation. If the initial study was not available, the authors were contacted to request access. A flowchart of the selected articles is shown in Figure 1.

### Inclusion and exclusion criteria

The selected studies met the following criteria: 1) published reports in English that examined the AMR of NG, excluding resistance to extended-spectrum cephalosporins and macrolides; 2) provided the sample size of assessed isolates; and 3) described AMR according to the standards of the Clinical and Laboratory Standards Institute (CLSI), the European Committee on Antimicrobial Susceptibility Testing (EUCAST), and/or WHO Resistance Surveillance Program and AGSP plans. The exclusion criteria for this study were as follows: 1) articles with duplicate data or overlapping studies and 2) *in vivo* studies, reviews, meta-analyses, and/or systematic reviews.

### Data extraction

Data collected for each study included the author, year of study, year of publication, geographical areas (continents/countries), a sample size of NG isolates, a sample size of resistant NG isolates, AST methods (disk diffusion, agar dilution, microbroth dilution, and E-test), and resistance guidelines (CLSI, EUCAST, and WHO). The data were collected by two reviewers and verified by a third reviewer.

### Quality assessment

The quality of the studies included in this review was assessed by two reviewers using an adapted approach of the Newcastle–Ottawa assessment scale for cross-sectional studies (Modesti et al., 2016). A scoring system ranging from 0 to 7 was used to assess the quality of each study. Studies of high, moderate, and low quality received ≥ 6 points, 4–5 points, and ≤3 points, respectively. A higher score indicates a higher-quality study. A third reviewer was involved in cases of discrepancies.

### Statistical analysis

The meta-analysis was performed using a random-effects model and the meta-prop command (Duval and Tweedie, 2000) in R statistical software (R Foundation for Statistical Computing, Vienna, Austria) (Kubanova et al., 2010). This analysis included all prevalence statistics and their corresponding 95% confidence intervals.

The weighted pooled resistance (WPR) analysis method is a statistical technique employed to combine and analyze data from multiple studies or sources while accounting for differences in sample sizes, variances, and other factors. This method assigns weights to each study or data source based on their sample size, variance, or other criteria, such as methodological quality. The primary objective of the WPR analysis is to provide more precise and reliable estimates by giving more influence to studies with larger sample sizes, lower variance, or higher quality. In our study, we applied the WPR analysis method to calculate the overall antibiotic resistance rates by combining data from various research studies conducted in different regions, using different methodologies, and with varying sample sizes. By assigning weights to these studies, we aimed to generate a more accurate and comprehensive picture of antibiotic resistance patterns, accounting for the heterogeneity across studies. The WPR analysis allowed us to identify potential antibiotic resistance trends and patterns that might not be apparent when analyzing individual studies separately. Additionally, this approach helped us account for possible biases and discrepancies in the data, ensuring a more reliable representation of global antibiotic resistance patterns. We believe that providing a clear description of the WPR analysis approach will enhance the clarity and transparency of our methods, enabling readers to better understand and interpret our findings.

The I2 values (25, 50, and 75%) indicated low, medium, and high heterogeneity, respectively. Meta-regression models were used to analyze the changes in AMR over time. To analyze the evolution of antibiotic resistance over time, we conducted a meta-regression analysis using surveillance data from Australia, the United States, and China.

Publication bias was assessed using the Egger and Begg tests. The trim-and-Fill method is a simple, non-parametric approach that utilizes funnel plots to identify and adjust for potential publication bias in this meta-analysis (Duval and Tweedie, 2000). The fill-and-trim method mitigates potential biases arising from studies with small sample sizes, helping ensure a more accurate and reliable estimate of the overall effect.

### Study outcomes

The primary outcome of the study was the WPR rate of NG for various antibiotics, including ciprofloxacin, spectinomycin, trimethoprim/sulfamethoxazole, gentamicin, kanamycin, tetracycline, and chloramphenicol. A subgroup analysis was performed to examine several factors, including the year of publication (1988–2013, 2014–2018, and 2019–2021), geographical area (continents/countries), AST, and interpretation of resistance.

## Results

### Systematic literature search

In the initial search, 2350 reports were identified. After removing 125 duplicates, 2225 unique reports remained. Upon title and abstract screening, 1945 reports were excluded. For a detailed breakdown of the exclusion criteria and the list of excluded reports, please refer to Supplementary Table. After a full-text review, additional 55 reports were excluded. A total of 225 reports published between 1988 and 2021 were deemed eligible for the meta-analysis. Please refer to the Supplementary Table for a detailed list of these reports and their respective information. The PRISMA flowchart in Figure 1 provides a graphical representation of the study selection process.

### Characteristics of the included studies

**FIGURE 2:**
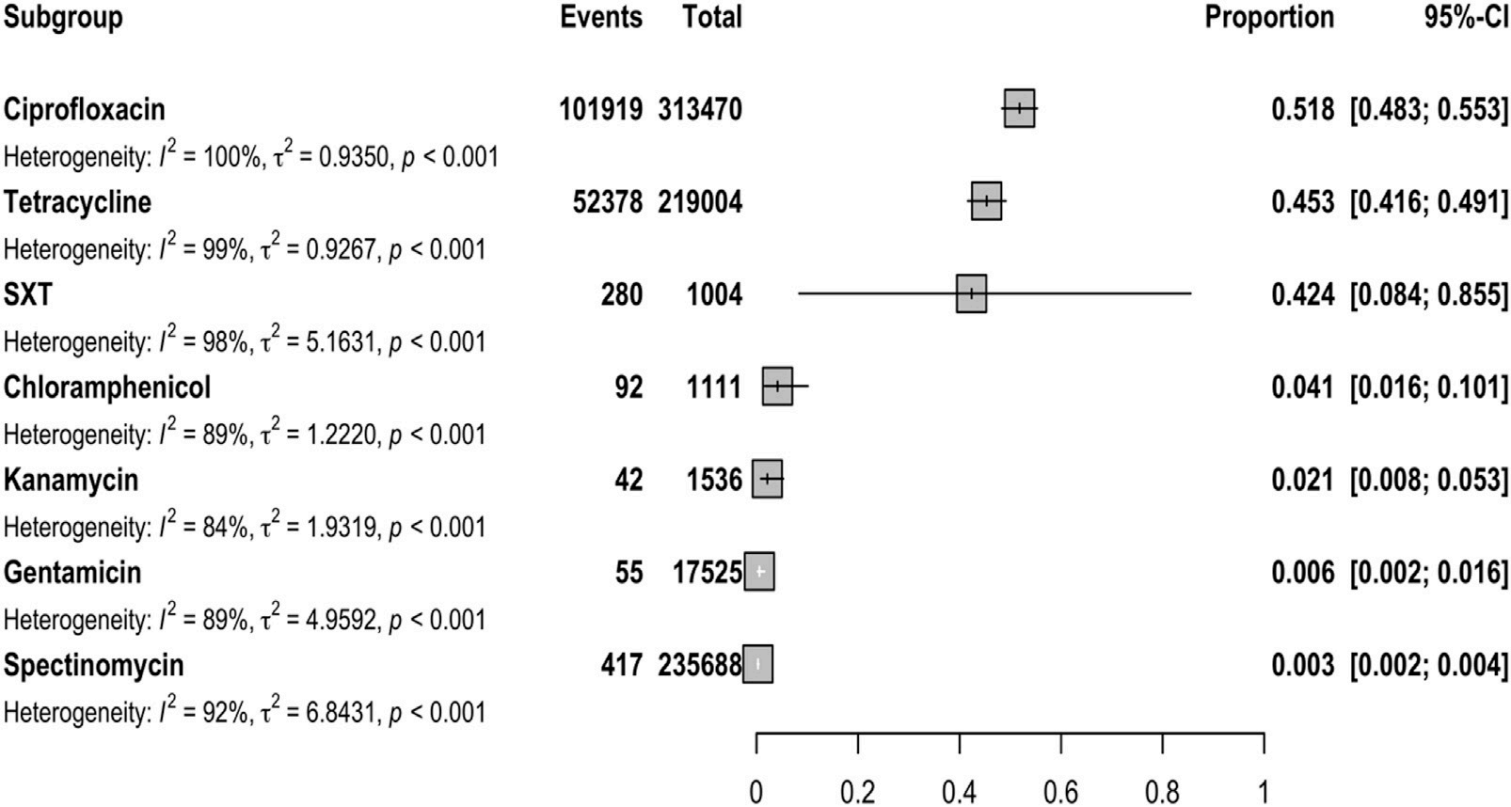
Forest plot summarizing antibiotic resistance rates. SXT, trimethoprim/sulfamethoxazole.

The meta-analysis included 225 reports from 68 countries. Most of the reports included in the study indicated resistance to ciprofloxacin and spectinomycin, followed by tetracycline (171 studies), gentamicin (24 studies), kanamycin (12 studies), chloramphenicol (8 studies), and trimethoprim/sulfamethoxazole (five studies). The forest plot (Figure 2) shows the proportion of isolates that are resistant to certain antibiotics. A high level of resistance to ciprofloxacin (51.6%), tetracycline (45.4%), and trimethoprim/sulfamethoxazole (42.4%) was observed. The rates of the individual antibiotics and the subgroup analyses are shown in Table 1.

**TABLE 1:** Subgroup analysis of the antibiotic resistance rates.

Antibiotic	Subgroup	Number of resistant isolates/number of evaluated isolates	Prevalence (%) of resistance (95% CI)	*p*-value
Spectinomycin		417/235,688	0.3 (0.2–0.4)	
	Continent			<0.01
	North America	102/58,872	0.00 (0.00–4)	
	Asia	110/35,357	1 (0.00–1)	
	Africa	80/3,064	1 (0.00–4)	
	Europe	80/31,921	0.00 (0.00–0.00)	
	South America	59/743	1 (0.00–16)	
	Oceania	0/214,028	0.00 (0.00–0.00)	
	Interpretation of resistance			
	CLSI	324/93,510	0.6 (0.3–1.00)	
	EUCAST	70/26,494	0.3 (0.1–0.8)	
	WHO	37/13,085	0.4 (0.2–0.6)	
	AGSP	0/16,1750		
	AST			0.01
	MIC-based methods	377/343,856	0.2 (0.1–0.4)	
	Disk diffusion	35/1,852	2.1 (0.5–8)	
	Mix methods	19/1,895	1.4 (0.5–3.5)	
Tetracycline		52,371/218,976	45.4 (41.7–49.2)	
	Continent			<0.01
	North America	17,787/87,614	26 (21–32)	
	Asia	10,791/15,870	64 (56–71)	
	Africa	1,534/1,961	86 (80–91)	
	Europe	7,987/26,365	26 (21–32)	
	South America	2,414/5,557	57 (46–67)	
	Oceania	25,739/179,866	13 (12–15)	
	Interpretation of resistance			
	CLSI	29,752/114,025	53.5 (48.4–58.6)	
	EUCAST	8,689/19,255	49.7 (44–55.4)	
	WHO	3,242/3,715	79.7 (67.8–88)	
	AGSP	20,572/161,742		
	AST			
	MIC-based methods	65,440/315,150	43.1 (39.3–47)	
	Disk diffusion	1,389/2,475	80.3 (61.2–91.3)	
	Mix methods	658/1,960	36.8 (20.1–57.4)	
Ciprofloxacin		101,891/313,442	51.6 (48.1–55.1)	
	Continent			<0.01
	North America	13,472/90,686	17 (12–23)	
	Asia	24,804/33,066	83 (78–88)	
	Africa	1,841/3,185	48 (34–63)	
	Europe	21,413/48,015	44 (41–47)	
	Oceania	58,250/216,716	24 (21–26)	
	South America	2,883/5,445	36 (26–48)	
	Interpretation of resistance			
	CLSI	133,831/35,587	52.6 (45.9–59.1)	
	EUCAST	65,143/32,543	52.2 (49.1–55.4)	
	WHO	12,741/8,562	79.5 (63.7–89.6)	
	AGSP	161,750/41,898		
	AST			
	MIC-based methods	130,441/423,609	50.5 (46.8–54.1)	
	Disk diffusion	2,366/3,545	66.4 (48.2–80.8)	
	Mix methods	2,491/3,899	51.3 (32.5–68.6469.7)	
Gentamicin		56/17,525	0.6 (0.2–16)	
	Continent			<0.01
	North America	0/10,630	0.00 (0.00–2)	
	Asia	1/712	0.00 (0.00–2)	
	Africa	29/1,307	2 (0.00–9)	
	Europe	0/2,125	0.00 (0.00–1)	
	Oceania	0/5,336	0.00 (0.00–0.00)	
	Interpretation of resistance			
	CLSI	50/12,385	1.1 (0.3–4.4)	
	EUCAST	4/2,283	0.6 (0.3, 1.1)	
	WHO	2/2,857	0.07 (0.02, 0.8)	
	AST			
	MIC-based methods	56/9,123	0.4 (0.2–1.2)	
	Disk diffusion	18/5,022	3.1 (0.00–76.9)	
	Mix methods	0/6,237	51.3 (32.5–69.7)	
Kanamycin		42/1,536	2.1 (0.8–5.3)	
	Continent			0.09
	Asia	30/625	4 (1–17)	
	Africa	11/453	3 (1–7)	
	Europe	1/458	1 (0.00–2)	
	Interpretation of resistance			
	CLSI	28/671	4 (1–7)	
	EUCAST	4/604	0.00 (0.00–2)	
	WHO	10/261	3 (0.00–11)	
	AST			
	MIC-based methods	38/790,739	1.9 (0.7–5.2)	
	Disk diffusion	4/5,022	4 (1.5–10.1)	
	Mix methods	0/6,237	51.3 (32.5–69.7)	
Chloramphenicol		92/1,111	4.1 (1.6–10.1)	
TMP/SMX		280/1,004	42.2 (8.4–85.5)	

The data in Table 1, particularly the discrepancy between the reported resistance rates and the cumulative data for tetracycline, among others, arise from the application of the weighted pooled analysis method described above. The reported resistance rate of 45% is a result of this weighted analysis, which might not directly correspond to the simple proportion (24%), calculated from the cumulative data (52,371 resistant samples out of 218,976 tested). The apparent discrepancy in the number of samples tested (218,976) *versus* the total derived from the methods (MIC + diffusion + mix, totaling 319,585) can be attributed to the fact that some samples were analyzed using more than one method. This multi-method analysis was essential for a comprehensive understanding of resistance patterns but resulted in a higher cumulative count of methods than the actual number of samples. This does not imply testing more samples than are available but rather reflects the multiple analyses conducted on the same sample set. Finally, the differences in sample numbers across continents and methods stem from the careful and detailed approach taken to analyze the data, considering the nuances of each study’s methodology and the availability of geographic information. This comprehensive approach, although complex, was necessary to provide the most accurate and informative analysis of antibiotic resistance patterns.

**FIGURE 3:**
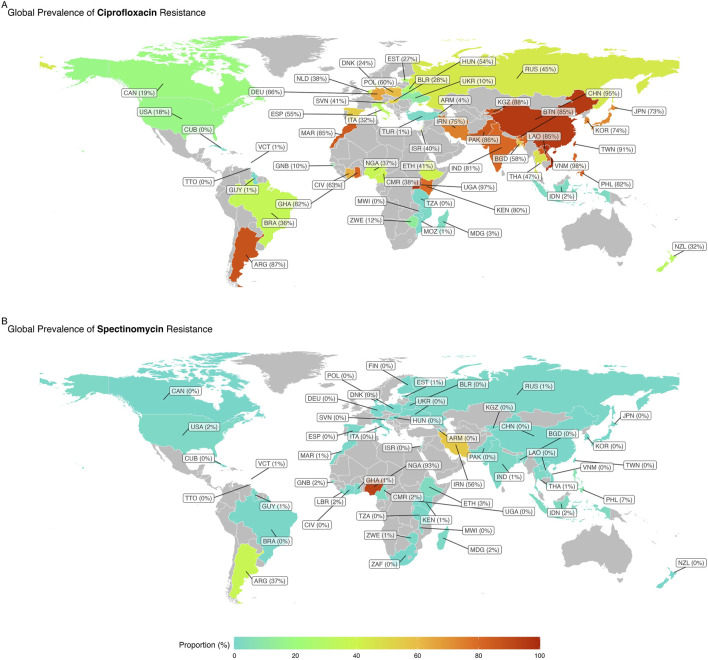
Global map of reported weighted pooled resistance rates for spectinomycin **(A)** and ciprofloxacin **(B)**.

Supplementary Figure S1 shows the trend in resistance to ciprofloxacin, spectinomycin, and tetracycline over time in Australia, the United States, and China, respectively. Figure 3 depicts the global map of reported WPR rates for spectinomycin and ciprofloxacin. The data on publication bias are shown in Table 2. Funnel plots are used to visually assess and depict potential publication bias in meta-analyses of studies on antibiotic resistance (Supplementary Figure S2). Resistance rates to various antibiotics are summarized as follows.

**TABLE 2:** Evaluation of publication bias in meta-analysis.

Antibiotic	Egger test	Begg test	Fail and safe	Trim and fill
Ciprofloxacin	*p* = 0.004	*p* = 0.009	245763	0.518 (0.483, 0.553)
SXT	*p* = 0.119	*p* = 0.817	0	0.424 (0.084, 0.855)
Gentamicin	*p* < 0.001	*p* = 0.007	4497	0.015 (0.006, 0.036)
Kanamycin	*p* < 0.001	*p* = 0.113	1341	0.045 (0.020, 0.097)
Tetracycline	*p* < 0.001	*p* = 0.523	503028	0.428 (0.391, 0.465)
Chloramphenicol	*p* < 0.001	*p* = 0.548	719	0.086 (0.037, 0.184)
Spectinomycin	*p* < 0.001	*p* < 0.001	268485	0.009 (0.006, 0.013)

This table provides a comprehensive assessment of potential publication bias in the meta-analysis using a range of statistical techniques. Included are statistics generated from Egger’s Method, Begg’s Method, the Fail-Safe N (NFS), and the trim-and-fill method. These methods are applied to investigate the presence of bias and its impact on the meta-analysis results, ensuring the robustness and reliability of the findings.

### Spectinomycin resistance

Spectinomycin resistance was reported in 171 studies with 235,688 NG isolates. The WPR rate was 0.3% (95% CI: 0.2%–0.4%), with significant heterogeneity (I2 = 92.03%; *p* < 0.01) (Table 1; Figure 2). Additionally, significant publication bias was found (*p* = 0.0001). Meta-regression analysis was performed to investigate temporal changes in the rate of spectinomycin resistance (Supplementary Figure S1). Meta-regression analysis showed a decrease in the resistance rate over time (r = −0.034, 95% CI: −1.192 to 0.233, and *p* = 0.233). Of the 56 countries reporting spectinomycin resistance rates, four countries (Nigeria, Argentina, Iran, and the Philippines) reported spectinomycin resistance rates above 5% (Figure 3). Significant differences in spectinomycin resistance rates were observed between countries (*p* < 0.01) (see Supplementary Figure S2). Meta-regression analysis showed that the rate of spectinomycin resistance decreased over time in Australia (r = −0.040; 95% CI: −0.094 to 0.015), the United States (r = −0.431; 95% CI: −0.758 to −0.105), and China (r = −0.125; 95% CI: −0.209 to −0.041) (see Supplementary Figure S1). A significant difference in spectinomycin resistance rates was observed between continents (*p* < 0.01), with higher rates in Asia, Africa, and South America than in other continents (1% vs 0%). Significant changes were observed in AST levels and resistance interpretations (*p* = 0.01) (Table 1). Implementing the fill-and-trim method yielded a proportion of 0.009 (95% CI: 0.006–0.013). No study exceeded the 3.622 threshold for studentized residuals, indicating no outliers. However, Cook’s distances identified potentially influential studies (Putnam et al., 1992; Ehinmidu et al., 2004; Kubanova et al., 2010; Azizmohammadi and Azizmohammadi, 2016). After their removal, the proportion remained at 0.009 (95% CI: 0.006–0.013). Funnel plot asymmetry was indicated by the rank correlation test (*p* < 0.001) but not the regression test (*p* = 0.130) (Supplementary Figure S2).

### Tetracycline resistance

Tetracycline resistance was reported in 171 studies with 218,976 NG isolates. The WPR rate was 45.4% (95% CI: 41.7%–49.2%), with significant heterogeneity (I2 = 99.39%) (Table 1; Figure 2). Additionally, significant publication bias was found (*p* = 0.0001). Meta-regression analysis revealed a significant increase in the tetracycline resistance rate over time (r = 0.035; 95% CI: 0.017 to 0.052; and *p* < 0.001) (Supplementary Figure S1). Among the 60 countries that reported resistance data for tetracycline, 32 (66.6%), including Ghana, Côte d'Ivoire, Brazil, Cameroon, Bhutan, Laos, the Philippines, Argentina, Pakistan, Madagascar, Malawi, Mozambique, Zimbabwe, Morocco, Liberia, Cuba, Guyana, Slovenia, Thailand, Uganda, Guinea-Bissau, Ethiopia, Gambia, Iran, Hungary, Korea, Indonesia, Kenya, Vietnam, Bangladesh, China, and Nigeria, reported tetracycline resistance in more than 45% of isolates. There was a significant change in tetracycline resistance rates among the different countries (*p* < 0.0001) (see Supplementary Figure S2). Meta-regression analysis showed that the rate of tetracycline resistance decreased over time in both the United States (r = −0.032; 95% CI: −0.054 to −0.010) and China (r = −0.020; 95% CI: −0.209 to −0.041). However, the rate of tetracycline resistance in Australia increased significantly over time (r = 0.064; 95% CI: 0.051–0.077) (Supplementary Figure S1). There was a significant difference in tetracycline resistance rates among continents (*p* < 0.01). Africa has a higher rate than Asia (86% vs 64%), South America (86% vs 57%), and Europe and North America (86% vs 26%). Significant changes were observed in the AST and interpretation of resistance (*p* = 0.001) (Table 1).

Upon applying the fill-and-trim method, the calculated proportion was 0.428 with a 95% confidence interval of 0.391–0.465. Studentized residuals revealed several studies, including those by Ieven et al. (2003); Li et al. (2014); Phouangsouvanh et al. (2018); Rambaran et al. (2019), exceeded the 3.623 threshold, suggesting the presence of potential outliers. Excluding these outliers did not alter the proportion, which remained at 0.428 with a 95% confidence interval of 0.391–0.465. Cook’s distances indicated no excessively influential studies. The regression test showed evidence of funnel plot asymmetry (*p* < 0.001), whereas the rank correlation test did not (*p* = 0.641).

### Ciprofloxacin resistance

Ciprofloxacin resistance was reported in 217 reports with a total of 313,442 NG isolates. The WPR rate was 51.6% (95% CI: 48.1%–55.1%), with significant heterogeneity (I2 = 99.54%; *p* < 0.01) (Table 1; Figure 2). Additionally, a significant publication bias was found (*p* = 0.0094). Meta-regression analysis revealed a significant increase in the rate of ciprofloxacin resistance over time (r = 0.035; 95% CI: 0.017–0.052; and *p* < 0.001) (Supplementary Figure S1). Among the 58 countries that reported resistance rates for ciprofloxacin, 24 (41.3%) countries, namely, Kyrgyzstan, Ghana, Côte d'Ivoire, Bhutan, Laos, the Philippines, Argentina, Spain, Taiwan, Pakistan, Iran, Uganda, Poland, Hungary, Morocco, Kenya, Korea, Norway, Pakistan, Vietnam, Germany, India, Bangladesh, and China, reported ciprofloxacin resistance in more than 50% of isolates (Figure 3). There was a significant change in ciprofloxacin resistance rates among different countries (*p* < 0.01) (see Supplementary Figure S2). Meta-regression analysis showed a significant increase in ciprofloxacin resistance rates over time in Australia (r = 0.073; 95% CI: 0.054–0.091), the United States (r = 0.039; 95% CI: 0.015–0.063), and China (r = 0.180; 95% CI: 0.136–0.225) (*p* < 0.05) (Supplementary Figure S1). There was a significant difference in ciprofloxacin resistance rates among continents (*p* < 0.01). Asia had higher rates than Africa (83% vs 48%), Europe (83% vs 44%), South America (83% vs 36%), Oceania (83% vs 24%), and North America (83% vs 17%). Applying the fill-and-trim method yielded a proportion of 0.518 (95% CI: 0.483–0.553). Multiple studies, including those by Ison and Martin (1999); Ieven et al. (2003); Golparian et al. (2014); Li et al. (2014); Yu et al. (2017); Thakur et al. (2018); Qin et al. (2019); Yan et al. (2019); Adamson et al. (2020); Dong et al. (2020); Kakooza et al. (2021); Le et al. (2021), showed studentized residuals greater than 3.684, indicating potential outliers. With these outliers removed, the proportion remained at 0.518 (95% CI: 0.483–0.553). Cook’s distances identified Ison and Martin (1999) as overly influential. Funnel plot asymmetry was suggested by the rank correlation test (*p* = 0.010) but not the regression test (*p* = 0.161).

### Gentamicin resistance

Analysis of 24 studies on gentamicin resistance in NG (17,525 isolates) revealed a 0.6% WPR rate, significant heterogeneity (I^2^ = 89.24% and *p* < 0.01), and publication bias (*p* = 0.0001). Meta-regression analysis indicated a decline in resistance over time (r = −0.044, 95% CI: −0.185 to 0.097, and *p* = 0.542) (Supplementary Figure S1). Among the 15 countries that reported resistance to gentamicin, Nigeria and Kenya reported resistance in more than 5% of isolates (13.3% of countries). Significant differences in gentamicin resistance rates were observed between the different countries (*p* < 0.01) (see Supplementary Figure S2). A significant difference in gentamicin resistance rates was observed among continents (*p* < 0.01), with Africa having a higher rate than other continents (2% vs 0%) (Supplementary Figure S2). Implementing the fill-and-trim method resulted in a proportion of 0.015 (95% CI: 0.006–0.036). No studies showed studentized residuals exceeding 3.078, suggesting no outliers. Cook’s distances identified potentially influential studies by Kularatne et al. (2018); Mann et al. (2018); and Nacht et al. (2020). After removing these studies, the proportion remained 0.015 (95% CI: 0.006–0.036). Funnel plot asymmetry was indicated by the rank correlation test (*p* = 0.028) but not the regression test (*p* = 0.846).

### Kanamycin resistance

Kanamycin resistance was discussed in 12 studies and included 1,536 NG isolates. The WPR rate was 2.1% (95% CI: 0.8%–5.3%), with significant heterogeneity (I2 = 83.78% and *p* < 0.01) (Table 1; Figure 2). A significant publication bias was found (*p* = 0.0001). Meta-regression analysis indicated a decline in the rate of kanamycin resistance over time (r = −0.083, 95% CI: −0.17 to 0.005, and *p* = 0.063) (Supplementary Figure S1). Significant differences in kanamycin resistance rates were observed among different countries (*p* < 0.01) (see Supplementary Figure S2). The rate of kanamycin resistance was higher in Asia than in Africa (4% vs 3%) and Europe (4% vs 1%). Significant changes were observed in the AST values and the interpretation of resistance (*p* > 0.05). After applying the fill-and-trim method, the proportion was 0.045 (95% CI: 0.020–0.097). Knapp et al. (1997) showed a studentized residual exceeding 2.865, suggesting a potential outlier. Removing this study maintained the proportion at 0.045 (95% CI: 0.020–0.097). No studies were identified as overly influential using Cook’s distances. Funnel plot asymmetry was indicated by both the rank correlation and regression tests (*p* = 0.028 and *p* < 0.001, respectively).

### Chloramphenicol resistance

Resistance to chloramphenicol was discussed in eight reports and included 1,111 NG isolates. The WPR rate was 4.1% (95% CI: 1.6%–10.1%), with significant heterogeneity (I2 = 89.39% and *p* < 0.01) (Table 1; Figure 2). A significant publication bias was found (*p* = 0.0001). Meta-regression analysis revealed a significant increase in the rate of chloramphenicol resistance over time (r = 0.122, 95% CI: 0.011–0.233, and *p* = 0.03) (Supplementary Figure S1). There were significant differences in chloramphenicol resistance rates between different countries and continents (*p* < 0.01). South America has a higher resistance rate than Europe (12% vs 5%), Africa (12% vs 3%), and Asia (12% vs 1%). Significant changes were observed in AST levels and resistance (*p* > 0.05). Implementing the fill-and-trim method resulted in a proportion of 0.086 (95% CI: 0.037–0.184). No studies showed studentized residuals exceeding 2.734, indicating no outliers. Cook’s distances identified no overly influential studies. Funnel plot asymmetry was suggested by the regression test (*p* < 0.001) but not the rank correlation test (*p* = 0.548).

### Trimethoprim/sulfamethoxazole resistance

Resistance to trimethoprim/sulfamethoxazole was reported in five studies and included a total of 1004 NG isolates. The WPR rate was 42.2% (95% CI: 8.4%–85.5%) with significant heterogeneity (I2 = 97.69% and *p* < 0.01) (Table 1; Figure 2). Furthermore, there was no significant evidence of publication bias (*p* = 0.1193). Utilizing the fill-and-trim method resulted in a proportion of 0.424 with a 95% confidence interval of 0.084–0.855. Analysis of studentized residuals showed a potential outlier in the study conducted by Brett et al. (1992), with a value greater than 2.576. Excluding this study, the proportion stayed at 0.424 with a 95% confidence interval of 0.084–0.855. No studies were found to be overly influential based on Cook’s distances. Both the rank correlation and regression tests did not indicate funnel plot asymmetry (*p* > 0.999 and *p* = 0.175, respectively).

## Discussion

According to current evidence, there is no effective vaccine that provides strong protection against NG infections (Zhu et al., 2021). The effectiveness of infection control depends on the use of appropriate antibiotic therapies. The widespread resistance of NG isolates to multiple antibiotics is a global concern. The WHO-GASP recommends conducting epidemiological investigations to understand the spread and extent of AMR and effectively manage gonorrhea treatment programs. Therefore, it is crucial to comprehensively understand the AMR profile of NG to effectively address this significant health problem. Recent research has enhanced our knowledge of the worldwide prevalence of AMR in NG patients. Our review analyzed global trends in NG antimicrobial resistance.

This indicated that the proportion of isolates resistant to spectinomycin, gentamicin, and kanamycin decreased significantly over time.

Spectinomycin is an effective option to eliminate kanamycin-resistant isolates. The results of this meta-analysis showed that spectinomycin, with a WPR of 0.3%, was the most effective among the antibiotics investigated. It binds to the 30S subunit of the ribosome and inhibits protein synthesis (Lancaster et al., 2015). The efficacy of spectinomycin in the treatment of pharyngeal gonorrhea is low (51.8%) due to its pharmacokinetic (PK) properties (Moran, 1995). The first spectinomycin-resistant strain was reported in 1967 (Aitolo et al., 2021). Resistance to spectinomycin has been documented in various countries across Asia, America, and Africa. Mutations in 16S rRNA genes are thought to contribute significantly to spectinomycin resistance despite the exact mechanism being unclear (Galimand et al., 2000). These genetic mutations are believed to play a key role in developing resistance to this antibiotic (Lee et al., 2016; Aniskevich et al., 2021; Armstrong et al., 2021).

Tetracycline is a commonly used antibiotic for gonorrhea treatment (Shaskolskiy et al., 2018). Like spectinomycin, tetracycline inhibits protein synthesis by blocking its binding to the 30S ribosomal subunit (Kivata et al., 2020). In recent decades, this antibiotic has been widely used for the treatment of gonococcal infections. However, owing to the increasing number of tetracycline-resistant NG isolates, treatment failures have increased more frequently. According to a previous study, the AMR for tetracycline in Africa was 100% (Rambaran et al., 2019). Some reports have indicated high rates of tetracycline resistance in Iran and China, with prevalence rates of 71% and 59%, respectively (Azizmohammadi and Azizmohammadi, 2016; Yan et al., 2019). In this study, tetracycline resistance remained consistently high over time. Chromosomal mutations and the acquisition of plasmid-borne genes are the main mechanisms underlying tetracycline resistance (Kivata et al., 2020). The chromosomal mutation involved the substitution of the amino acids Val57Met or Val57Leu in S10, which is encoded by the *rpsJ* gene. This mutation in the tetracycline-binding site disrupts the ability of tetracycline antibiotics to effectively bind and inhibit protein synthesis, potentially leading to the development of antibiotic resistance. The acquisition of plasmids carrying tetM is both crucial and specific. This plasmid was first discovered in 1985 in the United States (Morse et al., 1986) and in 1991 in Holland (Gascoyne et al., 1991) but has since become widespread globally. The TetM protein binds to the 30S ribosomal subunit and inhibits the binding of antibiotics to their targets (Chopra and Roberts, 2001).

Our study revealed gonococcal resistance rates of 51.6% for ciprofloxacin and 42.2% for trimethoprim/sulfamethoxazole, indicating a rising trend in ciprofloxacin resistance over time. Ciprofloxacin was commonly used to treat NG infections in the mid-1980s due to its safety and efficacy (Anderson et al., 2003). Resistant isolates were first identified in the 1990s (Gransden et al., 1990). The rate of ciprofloxacin resistance has significantly increased over the years (Crucitti et al., 2020), likely due to overuse and misuse of the drug. In Benin, no ciprofloxacin-resistant isolates were found in studies conducted in 1998 and 1999 (van Dyck et al., 2001), but resistance rates increased to 75% between 2015 and 2017 (Affolabi et al., 2018).

Tayimetha et al. found a significant increase in ciprofloxacin resistance, from 3.8% in 2009 to 50.6% in 2014 (*p* < 0.05) (Tayimetha and Unemo, 2018), which is consistent with findings reported by Crucitti et al. (2020). In these studies, the primary mechanism responsible for ciprofloxacin resistance was also identified. Resistance to ciprofloxacin in *Neisseria* species is associated with specific single-nucleotide positions in the quinolone resistance-determining region (QRDR) of gyrA and parC (da Costa-Lourenço et al., 2018; Low and Unemo, 2016). Increasing resistance to ciprofloxacin has led to its removal from the CDC list of recommended treatments for gonococcal infections (Unemo and Shafer, 2014b). It is no longer recommended as a first-line therapy (Queirós et al., 2020). Therefore, spectinomycin is recommended for patients infected with ciprofloxacin-resistant strains.

Trimethoprim/sulfamethoxazole was introduced in the early 1950s (Golparian et al., 2020). However, resistant isolates are widespread globally (Naznin et al., 2018). Currently, the resistance rate to trimethoprim/sulfamethoxazole is high and stable.

Gentamicin is an aminoglycoside antimicrobial that has been used for gonococcal infections in many developing countries due to its cost-effectiveness, single-dose option, efficacy, and safety (Ross and Lewis, 2012; Riedel et al., 2019; Kularatne et al., 2020). Previous studies (Kirkcaldy et al., 2014; Liu et al., 2019; Riedel et al., 2019) suggested that gentamicin can effectively treat gonorrheal infections. However, more comprehensive preclinical and clinical data are required to determine the therapeutic efficacy of gentamicin for treating gonococcal infections.

Gentamicin is not currently recommended for use in developed countries. The scarcity of gentamicin in developed countries and difficulties encountered in many regions were the primary factors contributing to the low prevalence of gentamicin-resistant strains in our analysis. The MIC value for gentamycin was not determined by the CLSI. Due to the inconsistent results of agar dilution and E-test methods, there are concerns about the lack of a reliable breakpoint and method for detecting resistance.

Although kanamycin as an aminoglycoside continues to be used in Africa (Apalata et al., 2009) for the treatment of gonorrhea, there is a paucity of susceptibility data (Apalata et al., 2009).

This study had some limitations that needed to be considered. The results of the reports estimating the proportion may not accurately represent the global burden of AMR in the NG, and caution should be exercised when interpreting these findings. In some reports, the sample size of the NG isolates analyzed was small, resulting in low statistical power. Several reports have not provided clear information about the study population or the duration of data collection. Our meta-analysis included only published reports and did not analyze the primary data.

## Conclusion

The global increase of AMR in NG necessitates understanding its prevalence to effectively control gonococcal infections. Our study highlights a concerning trend: increasing resistance to tetracycline and ciprofloxacin, despite their disuse for gonorrhea treatment. This emphasizes the ongoing challenge of selective pressure from antibiotic use in other contexts. However, a positive finding emerged: resistance rates to spectinomycin (0.3%) are decreasing. Continuous, global monitoring of NG AMR profiles remains crucial to ensure appropriate treatment and prevent the spread of resistant strains.

## Data Availability

The original contributions presented in the study are included in the article/Supplementary Material; further inquiries can be directed to the corresponding authors.

## Abbreviations

AMR, antimicrobial resistance; AST, antimicrobial susceptibility testing; AGSP, Australian Gonococcal Surveillance Programme; CI, confidence interval; CLSI, Clinical and Laboratory Standards Institute; EUCAST, European Committee on Antimicrobial Susceptibility Testing; NG, *Neisseria gonorrhoeae;* PRISMA, Preferred Reporting Items for Systematic Reviews and Meta-Analyses; STI, sexually transmitted infection; WHO-GASP, WHO Gonococcal AMR Surveillance Programme; WPR, weighted pooled resistance rate.
