# Corrigendum: Global trends of antimicrobial resistance rates in *Neisseria gonorrhoeae*: a systematic review and meta-analysis

**DOI:** 10.3389/fphar.2024.1465628

**Published:** 2024-09-03

**Authors:** Mohammad Hosseini Hooshiar, Mohammad Sholeh, Masoumeh Beig, Khalil Azizian, Ebrahim Kouhsari

**Affiliations:** ^1^ Department of Periodontology, School of Dentistry, Tehran University of Medical Sciences, Tehran, Iran; ^2^ Department of Bacteriology, Pasteur Institute of Iran, Tehran, Iran; ^3^ Department of Microbiology, Faculty of Medicine, Kurdistan University of Medical Sciences, Sanandaj, Iran; ^4^ Zoonoses Research Center, Research Institute for Health Development, Kurdistan University of Medical Sciences, Sanandaj, Iran; ^5^ Laboratory Sciences Research Center, Golestan University of Medical Sciences, Gorgan, Iran; ^6^ Department of Laboratory Sciences, Faculty of Paramedicine, Golestan University of Medical Sciences, Gorgan, Iran

**Keywords:** *Neisseria gonorrhoeae*, antimicrobial resistance, systematic review and meta-analysis, spectinomycin, gonorrhea

In the published article, there was an error in Figure 3 as published. There was an inaccuracy in the information regarding ciprofloxacin resistance in New Zealand. The corrected Figure 3 and its caption appear below.

**FIGURE 3:**
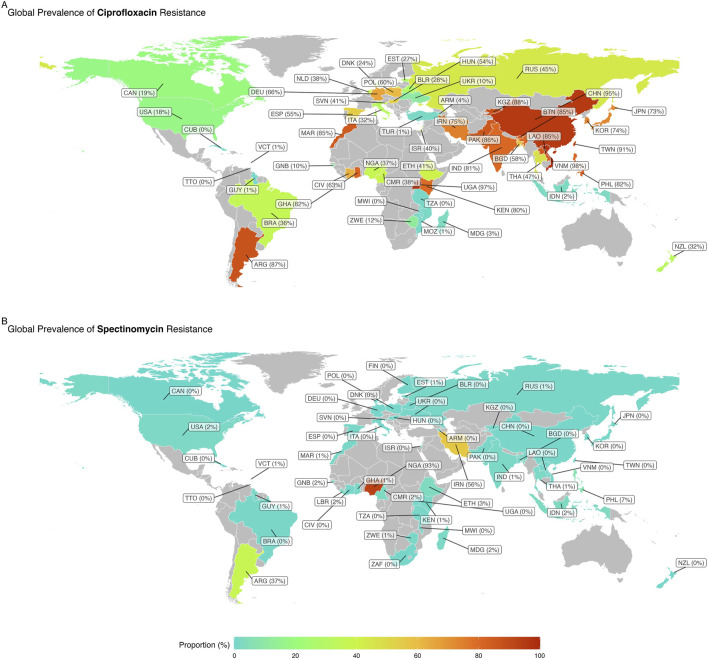
Global map of reported weighted pooled resistance rates for spectinomycin **(A)** and ciprofloxacin **(B)**.

In the published article, there was an error in **Supplementary Data Sheet 2**. In **Supplementary Figure S2** ‘Forest plot for the ciprofloxacin resistance rate stratified by countries’, data for New Zealand erroneously appears on 2 lines. The line indicating 126 ciprofloxacin resistant isolates out of 398 is the correct information. The second line (indicating 28 resistant isolates out of 28) is wrong and has been removed. The correct material statement appears below.

Supplementary Figure S2 | Forest plot for the ciprofloxacin resistance rate stratified by countries.supplementary-material.

In the published article, there was an error. Figure 3 has been updated to reflect that the correct data for New Zealand ciprofloxacin resistance is 126/398. This means that the correlating text related to Figure 3 must also be updated.

A correction has been made to **Results**, *Ciprofloxacin resistance*, Paragraph Number 1. This sentence previously stated:

“Among the 58 countries that reported resistance rates for ciprofloxacin, 25 (43.1%) countries, namely, Kyrgyzstan, Ghana, Côte d’Ivoire, Bhutan, Laos, the Philippines, Argentina, Spain, Taiwan, Pakistan, Iran, Uganda, Poland, Hungary, Morocco, Kenya, Korea, New Zealand, Norway, Pakistan, Vietnam, Germany, India, Bangladesh, and China, reported ciprofloxacin resistance in more than 50% of isolates (Figure 3).”

The corrected sentence appears below:

“Among the 58 countries that reported resistance rates for ciprofloxacin, 24 (41.3%) countries, namely, Kyrgyzstan, Ghana, Côte d’Ivoire, Bhutan, Laos, the Philippines, Argentina, Spain, Taiwan, Pakistan, Iran, Uganda, Poland, Hungary, Morocco, Kenya, Korea, Norway, Pakistan, Vietnam, Germany, India, Bangladesh, and China, reported ciprofloxacin resistance in more than 50% of isolates (Figure 3).”

The authors apologize for these errors and state that this does not change the scientific conclusions of the article in any way. The original article has been updated.

