# Supplementary material for: Global trends of antimicrobial resistance rates in Neisseria gonorrhoeae: a systematic review and meta-analysis
Source: Front Pharmacol. 2024 Jul 3;15:1284665. doi: 10.3389/fphar.2024.1284665 (PMC11258497; doi:10.3389/fphar.2024.1284665)
Supplement: Supplementary file 1 [file DataSheet3.docx]

| ID | First author | Published time | countries | AST | AST guidelines | No. isolates | ciprofloxacin | SXT | gentamicin | kanamycin | tetracycline | chloramphenicol | spectinomycin | QA | References |
| --- | --- | --- | --- | --- | --- | --- | --- | --- | --- | --- | --- | --- | --- | --- | --- |
| 11 | Thomas Fekete et al | 1989 | USA | MIC base | CLSI | 129 |  |  |  |  |  |  | 0 | 7 | (1) |
| 12 | Vivian G Loo et al | 1990 | Canada | MIC base | CLSI | 296 | 43 |  |  |  | 78 |  | 0 | 7 | (2) |
| 13 | Thomas Fekete et al | 1991 | USA | MIC base | CLSI | 77 |  |  |  |  | 39 |  |  | 4 | (3) |
| 15 | Shannon D. Putnam et al | 1992 | USA | MIC base | CLSI | 47 | 2 |  |  |  | 6 |  | 40 | 7 | (4) |
| 16 | Shannon D. Putnam et al | 1992 | USA | MIC base | CLSI | 71 | 14 |  |  |  | 45 |  | 62 | 7 | (4) |
| 23 | Roselyn J. Rice et al | 1994 | USA | MIC base | CLSI | 84 | 0 |  | 0 |  | 17 |  |  | 4 | (5) |
| 24 | Roselyn J. Rice et al | 1994 | USA | Mixed | CLSI | 62 | 0 |  |  |  |  |  |  | 4 | (6) |
| 25 | M.R. Joesef et al | 1994 | Indonesia | MIC base | CLSI | 86 | 0 |  | 0 |  | 84 | 0 | 16 | 7 | (7) |
| 26 | B West et al | 1995 | Tanzania | MIC base | WHO | 130 | 0 |  |  | 5 |  |  | 0 | 5 | (8) |
| 27 | S. Knapp et al | 1995 | Jamaica | MIC base | CLSI | 116 |  |  |  |  | 25 |  |  | 5 | (9) |
| 28 | Mariella C. Cummings et al | 1995 | USA | MIC base | CLSI | 324 |  |  |  |  | 46 |  | 0 | 6 | (10) |
| 29 | Mariella C. Cummings et al | 1995 | USA | MIC base | CLSI | 500 |  |  |  |  |  |  | 0 | 6 | (10) |
| 31 | Kai Man Kam et al | 1996 | China | MIC base | CLSI | 69 |  |  |  |  | 62 |  | 0 | 4 | (11) |
| 38 | Antti Nissinen et al | 1997 | Finland | MIC base | CLSI | 337 |  |  |  |  |  |  | 0 | 4 | (12) |
| 34 | R. A. Adegbola et al | 1997 | Gambia | Disc diffusion | CLSI | 111 |  | 109 |  |  | 100 | 0 |  | 6 | (13) |
| 36 | Joan S. Knapp et al | 1997 | Thailand | MIC base | CLSI | 101 | 1 |  |  | 22 |  |  | 0 | 7 | (14) |
| 40 | Andrea Guyot et al | 1998 | Liberia | MIC base | CLSI | 100 |  |  |  |  | 63 |  | 2 | 6 | (15) |
| 42 | Bahar Uddin Bhuiyan et al | 1999 | Bangladesh | MIC base | CLSI | 94 | 11 |  |  |  | 57 |  | 0 | 7 | (16) |
| 45 | Krishna Ray et al | 2000 | India | Disc diffusion | CLSI | 211 | 25 |  |  |  | 14 |  |  | 5 | (17) |
| 46 | Krishna Ray et al | 2000 | India | MIC base | CLSI | 55 | 12 |  |  |  | 10 |  |  | 5 | (17) |
| 44 | Cao Wenling et al | 2000 | China | MIC base | WHO | 203 | 123 |  |  |  |  |  | 0 | 6 | (18) |
| 51 | Jo-Anne R Dillon et al | 2001 | Brazil | MIC base | CLSI | 81 | 0 |  |  |  | 69 |  | 0 | 6 | (19) |
| 52 | Jo-Anne R Dillon et al | 2001 | Guyana | MIC base | CLSI | 70 | 0 |  |  |  | 62 |  | 0 | 6 | (19) |
| 53 | Jo-Anne R Dillon et al | 2001 | St. Vincent | MIC base | CLSI | 68 | 0 |  |  |  | 29 |  | 0 | 6 | (19) |
| 54 | Jo-Anne R Dillon et al | 2001 | Trinidad and Tobago | MIC base | CLSI | 144 | 0 |  |  |  | 57 |  | 0 | 6 | (19) |
| 56 | SHUNZHANG YE et al | 2002 | China | MIC base | WHO | 2549 | 873 |  |  |  |  |  |  | 7 | (20) |
| 57 | SHUNZHANG YE et al | 2001 | China | MIC base | WHO | 1060 |  |  |  |  | 976 |  |  | 7 | (20) |
| 59 | SHUNZHANG YE et al | 2001 | China | MIC base | WHO | 3175 |  |  |  |  |  |  | 14 | 7 | (20) |
| 69 | LAI-KING NG et al | 2002 | Canada | MIC base | CLSI | 2687 | 130 |  |  |  |  |  |  | 4 | (21) |
| 60 | Rafael Llanes et al | 2002 | Cuba | MIC base | CLSI | 120 |  |  |  |  | 65 |  |  | 6 | (22) |
| 63 | Shunzahng Ye et al | 2002 | China | MIC base | WHO | 2549 | 873 |  |  |  |  |  |  | 6 | (20) |
| 64 | Shunzahng Ye et al | 2002 | China | MIC base | WHO | 1060 |  |  |  |  | 986 |  |  | 6 | (20) |
| 66 | Shunzahng Ye et al | 2002 | China | MIC base | WHO | 3175 |  |  |  |  |  |  | 14 | 6 | (20) |
| 67 | David L. Trees et al | 2002 | Thailand | MIC base | WHO | 168 | 36 |  |  |  |  |  |  | 6 | (23) |
| 68 | Motiur Rahman et al | 2002 | Bangladesh | MIC base | CLSI | 311 | 94 |  |  |  | 212 |  | 0 | 6 | (24) |
| 61 | Margareta Ieven et al | 2002 | Indonesia | MIC base | CLSI | 267 | 0 |  |  | 0 | 264 |  | 0 | 7 | (25) |
| 70 | P Bhalla et al | 2002 | India | MIC base | CLSI | 44 | 40 |  |  |  | 1 |  |  | 7 | (26) |
| 72 | Michael Dan et al | 2002 | Israel | MIC base | CLSI | 100 | 61 |  |  |  | 8 |  | 0 | 7 | (27) |
| 77 | Jorge Sosa et al | 2003 | Cuba | MIC base | CLSI | 91 | 0 |  |  |  | 76 |  | 0 | 6 | (28) |
| 84 | Ehinmidu J O et al | 2004 | Nigeria | MIC base | CLSI | 57 | 21 |  | 6 |  | 57 |  | 53 | 4 | (29) |
| 82 | K Shigemura et al | 2004 | Japan | MIC base | CLSI | 87 | 60 |  |  |  | 17 |  | 0 | 5 | (30) |
| 81 | Jeongsik Yoo et al | 2004 | Korea | MIC base | CLSI | 817 | 161 |  |  |  | 469 |  | 0 | 6 | (31) |
| 85 | Derya Aydın et al | 2005 | Turkey | MIC base | CLSI | 78 | 1 |  |  |  | 14 |  |  | 6 | (32) |
| 87 | Yang Yang et al | 2006 | China | MIC base | CLSI | 159 | 157 |  |  |  | 90 |  | 0 | 4 | (33) |
| 86 | M. Enders et al | 2006 | Germany | MIC base | CLSI | 65 | 31 |  |  |  | 19 |  | 0 | 6 | (34) |
| 90 | Sunil Sethi et al | 2006 | India | MIC base | CLSI | 45 | 35 |  |  |  | 23 |  |  | 6 | (35) |
| 91 | Elizabeth A Donegan. et al | 2006 | Indonesia | MIC base | CLSI | 147 | 59 |  |  |  | 147 |  |  | 6 | (36) |
| 88 | Bei Wang et al | 2006 | China | MIC base | CLSI | 95 | 95 |  |  |  | 75 |  | 1 | 7 | (37) |
| 94 | P Khaki et al | 2007 | India | MIC base | CLSI | 60 | 59 |  |  |  | 12 |  | 0 | 6 | (38) |
| 96 | XIAOHONG SU et al | 2007 | China | MIC base | WHO | 1208 | 1167 |  |  |  |  |  | 7 | 6 | (39) |
| 98 | Veronica Vorobieva et al | 2007 | Russian | MIC base | WHO | 76 | 13 |  |  | 1 | 9 |  | 0 | 7 | (40) |
| 99 | Gayane Hovhannisyan et al | 2007 | Armenia | Disc diffusion | CLSI | 101 | 4 |  |  | 4 |  | 0 | 0 | 7 | (41) |
| 104 | M Bala et al | 2008 | India | MIC base | WHO | 26 | 20 |  |  |  |  |  | 1 | 5 | (42) |
| 100 | D A Lewis et al | 2008 | MC | MIC base | CLSI | 272 | 81 |  |  |  |  |  |  | 7 | (43) |
| 101 | Van Cao et al | 2008 | MC | MIC base | CLSI | 28 | 0 |  |  |  | 28 |  | 0 | 7 | (44) |
| 102 | Van Cao et al | 2008 | Madagascar | MIC base | CLSI | 68 | 2 |  |  |  | 64 |  | 1 | 7 | (44) |
| 103 | Van Cao et al | 2008 | Vietnam | MIC base | CLSI | 177 | 168 |  |  |  | 116 |  | 0 | 7 | (44) |
| 105 | S. Starnino et al | 2008 | Italy | MIC base | CLSI | 326 | 111 |  |  |  | 62 |  | 0 | 7 | (45) |
| 106 | J Lis-Tønder et al | 2009 | Denmark | MIC base | CLSI | 62 | 30 |  |  |  |  |  |  | 4 | (46) |
| 107 | Sheldon R. Morris et al | 2009 | USA | MIC base | CLSI | 79 |  |  |  |  | 55 |  |  | 6 | (47) |
| 108 | Teke Apalata et al | 2009 | Mozambique | MIC base | WHO | 55 | 0 |  |  | 4 | 42 |  |  | 6 | (48) |
| 111 | Lillian B. Brown et al | 2010 | Malawi | MIC base | CLSI | 100 | 0 |  | 0 | 0 | 77 |  | 0 | 5 | (49) |
| 112 | Monir Uddin Ahmed et al | 2010 | Bangladesh | MIC base | CLSI | 1766 | 1412 |  |  |  | 1516 |  | 1 | 5 | (50) |
| 115 | Michael Dan et al | 2010 | Israel | MIC base | CLSI | 406 | 88 |  |  |  | 62 |  | 0 | 6 | (51) |
| 116 | A Kubanova et al | 2010 | Russian | MIC base | CLSI | 1560 | 769 |  |  |  |  |  | 71 | 7 | (52) |
| 119 | Masatoshi Tanaka et al | 2011 | Japan | MIC base | CLSI | 242 | 171 |  |  |  | 68 |  | 0 | 4 | (53) |
| 117 | Slavyana Glazkova et al | 2011 | Belarus | MIC base | EUCAST | 80 | 28 |  | 0 |  | 21 |  | 0 | 5 | (54) |
| 123 | Vanessa G. Allen et al | 2011 | Canada | MIC base | CLSI | 149 | 42 |  |  |  | 38 |  | 0 | 6 | (55) |
| 120 | Supriya D. Mehta et al | 2011 | Kenya | MIC base | CLSI | 105 | 13 |  |  |  | 102 |  | 0 | 7 | (56) |
| 127 | Birgitta Olsen et al | 2012 | Guinea-Bissau | Mixed | EUCAST | 31 | 3 |  |  |  | 23 |  | 0 | 5 | (57) |
| 128 | Irene Martin et al | 2012 | Canada | MIC base | CLSI | 155 | 6 |  |  |  |  |  | 0 | 5 | (58) |
| 126 | S. O. Hjelmevoll et al | 2012 | Norway | MIC base | EUCAST | 114 | 89 |  |  |  |  |  | 0 | 6 | (59) |
| 130 | Janice Yee Chi Lo et al | 2012 | China | MIC base | CLSI | 8123 |  |  |  |  |  |  | 0 | 6 | (60) |
| 147 | Ryoichi Hamasuna et al | 2013 | Japan | MIC base | CLSI | 83 | 65 |  |  |  |  |  | 0 | 4 | (61) |
| 144 | Robert D. Kirkcaldy et al | 2013 | USA | MIC base | CLSI | 34600 | 4252 |  |  |  | 6526 |  |  | 5 | (62) |
| 137 | S. SOOD et al | 2013 | India | MIC base | CLSI | 18 | 16 |  |  |  | 7 |  | 0 | 6 | (63) |
| 138 | Ken Shimuta et al | 2013 | Japan | MIC base | CLSI | 193 | 151 |  |  |  |  |  | 0 | 6 | (64) |
| 139 | Sunil Sethi et al | 2013 | MC | MIC base | EUCAST | 65 | 61 |  |  |  | 36 |  | 0 | 6 | (65) |
| 148 | Jo-Anne R Dillon et al | 2013 | MC | MIC base | CLSI | 12730 | 1884 |  |  |  |  |  |  | 6 | (66) |
| 149 | Jo-Anne R Dillon et al | 2013 | MC | MIC base | CLSI | 1913 |  |  |  |  | 832 |  |  | 6 | (66) |
| 141 | Birgitta Olsen et al | 2013 | Vietnam | MIC base | CLSI | 108 | 106 |  |  |  | 89 |  | 0 | 7 | (67) |
| 142 | Irene Martin et al | 2013 | Canada | MIC base | CLSI | 7428 | 1125 |  |  |  |  |  | 0 | 7 | (68) |
| 143 | Irene Martin et al | 2013 | Canada | MIC base | CLSI | 7205 |  |  |  |  | 1638 |  | 0 | 7 | (68) |
| 145 | Lígia Maria Bedeschi Costa et al | 2013 | Brazil | MIC base | CLSI | 201 | 43 |  |  |  | 65 | 24 | 0 | 7 | (69) |
| 146 | Amina Hançali et al | 2013 | Morocco | MIC base | CLSI | 72 | 61 |  |  |  | 66 |  | 0 | 7 | (70) |
| 153 | Robert D Kirkcaldy et al | 2013 | USA | MIC base | CLSI | 37233 | 4840 |  |  |  |  |  | 0 | 7 | (62) |
| 154 | Robert D Kirkcaldy et al | 2013 | USA | MIC base | CLSI | 27056 |  |  |  |  | 3850 |  |  | 7 | (62) |
| 178 | G La Ruche et al | 2014 | France | MIC base | EUCAST | 7688 |  |  |  |  | 3413 |  |  | 4 | (71) |
| 180 | G La Ruche et al | 2014 | France | MIC base | EUCAST | 8550 |  |  |  |  |  |  | 0 | 4 | (71) |
| 181 | G La Ruche et al | 2014 | France | MIC base | EUCAST | 8648 | 3413 |  |  |  |  |  |  | 4 | (71) |
| 158 | ÉVA NEMES-NIKODÉM et al | 2014 | Hungary | MIC base | EUCAST | 52 | 29 |  |  |  | 49 |  | 0 | 5 | (72) |
| 165 | Sai Li et al | 2014 | China | MIC base | CLSI | 334 | 330 |  |  |  | 327 |  | 0 | 5 | (73) |
| 166 | Wei-Ming Gu et al | 2014 | China | MIC base | CLSI | 3036 | 2590 |  |  |  |  |  |  | 5 | (74) |
| 167 | Wei-Ming Gu et al | 2014 | China | MIC base | CLSI | 1798 |  |  |  |  | 910 |  |  | 5 | (74) |
| 168 | Wei-Ming Gu et al | 2014 | China | MIC base | CLSI | 3153 |  |  |  |  |  |  | 2 | 5 | (74) |
| 169 | Samo Jeverica et al | 2014 | Slovenia | MIC base | EUCAST | 194 | 80 |  |  |  | 93 |  | 0 | 5 | (75) |
| 170 | Nicole Nari Horn et al | 2014 | Germany | MIC base | EUCAST | 213 | 157 |  |  |  | 88 |  | 0 | 5 | (76) |
| 157 | D. Golparian et al | 2014 | Estonia | MIC base | EUCAST | 44 | 12 |  |  |  | 15 |  | 0 | 6 | (77) |
| 171 | Beata Mlynarczyk-Bonikowska et al | 2014 | Poland | MIC base | EUCAST | 228 | 138 |  |  |  | 98 |  | 0 | 6 | (78) |
| 172 | Shao-Chun Chen et al | 2014 | China | MIC base | EUCAST | 278 | 278 |  |  |  |  |  | 0 | 6 | (79) |
| 173 | Alexandra Brunner et al | 2014 | Hungary | MIC base | EUCAST | 582 | 384 |  |  |  | 499 |  | 0 | 6 | (80) |
| 159 | Bang-yong Zhu et al | 2014 | China | MIC base | WHO | 923 | 857 |  |  |  |  |  | 1 | 7 | (81) |
| 160 | Heping Zheng et al | 2014 | China | MIC base | WHO | 1257 | 1157 |  |  |  |  |  | 0 | 7 | (82) |
| 161 | Judith Vandepitte et al | 2014 | Uganda | MIC base | EUCAST | 148 | 123 |  |  |  | 144 |  | 0 | 7 | (83) |
| 162 | Simbarashe Takuva et al | 2014 | Zimbabwe | MIC base | EUCAST | 66 | 4 |  |  | 0 |  |  |  | 7 | (84) |
| 163 | Simbarashe Takuva et al | 2014 | Zimbabwe | MIC base | EUCAST | 51 |  |  | 0 |  | 46 |  | 0 | 7 | (84) |
| 189 | S. W. Peterson et al | 2015 | Canada | MIC base | CLSI | 252 | 155 |  |  |  |  |  |  | 4 | (85) |
| 195 | Michelle J. Cole et al | 2015 | MC | MIC base | EUCAST | 1994 | 1055 |  |  |  |  |  | 0 | 4 | (86) |
| 183 | Fernando Cobo et al | 2015 | Spain | MIC base | EUCAST | 65 | 42 |  |  |  | 40 |  | 0 | 5 | (87) |
| 184 | Fernando Cobo et al | 2015 | Spain | MIC base | CLSI | 65 | 42 |  |  |  | 40 |  | 0 | 5 | (87) |
| 185 | Fiodar Lebedzeu et al | 2015 | Belarus | MIC base | EUCAST | 193 | 54 |  |  |  | 70 |  | 0 | 6 | (88) |
| 187 | Masatoshi Tanaka et al | 2015 | Japan | MIC base | CLSI | 677 | 480 |  |  |  | 115 |  | 0 | 6 | (89) |
| 190 | Hyukmin Lee et al | 2015 | Korea | MIC base | EUCAST | 210 | 204 |  |  |  | 105 |  | 0 | 7 | (90) |
| 192 | Ryoichi Hamasuna et al | 2015 | Japan | MIC base | CLSI | 103 | 81 |  |  |  |  |  | 0 | 7 | (91) |
| 193 | T Bharara et al | 2015 | India | Mixed | CLSI | 135 | 86 |  |  |  | 36 |  | 0 | 7 | (92) |
| 198 | Alexey Kubanov et al | 2016 | Russian | MIC base | EUCAST | 124 | 50 |  |  |  | 21 |  | 0 | 5 | (93) |
| 209 | Rasheda Khanam et al | 2016 | Bangladesh | Disc diffusion | CLSI | 21 | 20 |  |  |  |  |  | 0 | 5 | (94) |
| 215 | Susan Azizmohammadi et al | 2016 | Iran | Disc diffusion | CLSI | 32 | 24 |  |  |  | 23 | 1 | 18 | 5 | (95) |
| 196 | Anna Carannante et al | 2016 | Italy | MIC base | CLSI | 65 | 0 |  |  |  |  |  |  | 6 | (96) |
| 197 | Irene Fuertes de Vegaa et al | 2016 | Spain | MIC base | EUCAST | 110 | 58 |  | 0 |  |  |  | 1 | 6 | (97) |
| 199 | Alexandra Brunner et al | 2016 | Hungary | MIC base | EUCAST | 192 | 76 |  |  |  | 134 |  | 0 | 6 | (98) |
| 200 | J. Serra-Pladevall et al | 2016 | Spain | MIC base | EUCAST | 329 | 162 |  |  |  |  |  | 0 | 6 | (99) |
| 203 | Chanwit Tribuddharat et al | 2016 | Thailand | MIC base | CLSI | 350 |  |  |  |  | 312 |  |  | 6 | (100) |
| 204 | Chanwit Tribuddharat et al | 2016 | Thailand | MIC base | CLSI | 30 |  |  |  |  |  |  | 0 | 6 | (100) |
| 205 | Chanwit Tribuddharat et al | 2016 | Thailand | MIC base | CLSI | 349 | 307 |  |  |  |  |  |  | 6 | (100) |
| 208 | T Regnath et al | 2016 | Germany | MIC base | EUCAST | 434 | 319 |  |  |  | 210 |  | 0 | 6 | (101) |
| 210 | Kauser Jabeen et al | 2016 | Pakistan | MIC base | CLSI | 100 | 86 |  |  |  | 51 |  | 0 | 6 | (102) |
| 212 | T Regnath et al | 2016 | Germany | MIC base | CLSI | 434 | 280 |  |  |  | 210 |  | 0 | 6 | (101) |
| 207 | Yu-Hua Lin et al | 2016 | Taiwan | Mixed | CLSI | 202 | 183 |  |  |  |  |  |  | 7 | (103) |
| 211 | Ching-Wai Cheng et al | 2016 | Taiwan | Disc diffusion | CLSI | 1090 | 909 |  |  |  |  |  |  | 7 | (104) |
| 220 | Rui-xing Yu et al | 2017 | China | MIC base | WHO | 244 | 243 |  |  |  |  |  | 0 | 5 | (105) |
| 227 | P. R. S. Lagace´-Wiens et al | 2017 | Canada | MIC base | CLSI | 112 | 69 |  |  |  | 95 |  | 0 | 5 | (106) |
| 216 | J. Serra-Pladeval et al | 2017 | spain | MIC base | EUCAST | 111 | 60 |  |  |  |  |  |  | 6 | (107) |
| 217 | LI Wei et al | 2017 | China | MIC base | WHO | 303 | 293 |  |  |  |  |  | 0 | 6 | (108) |
| 229 | Ricardo Gianecini et al | 2017 | Argentina | MIC base | CLSI | 44 | 43 |  |  |  | 42 |  |  | 6 | (109) |
| 230 | Clark Martin P. Araneta et al | 2017 | Philippines | Disc diffusion | CLSI | 86 |  |  |  |  |  |  | 6 | 6 | (110) |
| 232 | Clark Martin P. Araneta et al | 2017 | Philippines | Disc diffusion | CLSI | 82 | 64 |  |  |  |  |  |  | 6 | (110) |
| 233 | Clark Martin P. Araneta et al | 2017 | Philippines | Disc diffusion | CLSI | 78 |  |  |  |  | 58 |  |  | 6 | (110) |
| 234 | Clark Martin P. Araneta et al | 2017 | Philippines | Disc diffusion | CLSI | 86 |  |  |  |  |  |  | 6 | 6 | (110) |
| 218 | Ahmed S Latif et al | 2017 | Zimbabwe | MIC base | CLSI | 102 | 19 |  |  | 2 |  |  |  | 7 | (111) |
| 222 | Ting Peng et al | 2017 | China | Mixed | CLSI | 1249 | 1177 |  |  |  |  |  |  | 7 | (112) |
| 223 | Seema Sood et al | 2017 | India | MIC base | CLSI | 204 | 193 |  |  |  |  |  |  | 7 | (113) |
| 228 | Fa-Xing Jiang et al | 2017 | China | MIC base | CLSI | 126 | 126 |  |  |  | 103 |  | 0 | 7 | (114) |
| 261 | Sanne HI Hofstraat et al | 2018 | Netherlands | MIC base | EUCAST | 2521 | 938 |  |  |  |  |  |  | 4 | (115) |
| 237 | Susanne Buder et al | 2018 | Germany | MIC base | EUCAST | 537 | 349 |  |  |  |  |  |  | 5 | (116) |
| 247 | Laura M. Mann et al | 2018 | USA | MIC base | CLSI | 10403 |  |  | 0 |  |  |  |  | 5 | (117) |
| 259 | Simon R Harris et al | 2018 | MC | MIC base | EUCAST | 1504 | 562 |  |  |  |  |  | 0 | 5 | (118) |
| 243 | Carolle Y. Tayimetha et al | 2018 | Cameroon | Disc diffusion | CLSI | 198 | 35 |  |  |  | 116 | 14 | 5 | 6 | (119) |
| 246 | Johan H. Melendez et al | 2018 | USA | MIC base | CLSI | 143 | 63 |  | 0 |  | 15 |  |  | 6 | (120) |
| 248 | Yen-Hung Liu et al | 2018 | Taiwan | MIC base | CLSI | 266 | 244 |  |  |  |  |  | 1 | 6 | (121) |
| 251 | Ranmini Kularatne et al | 2018 | South Africa | MIC base | EUCAST | 854 |  |  |  |  |  |  | 0 | 6 | (122) |
| 371 | Savandalath Phouangsouvanh et al | 2018 | Laos | Disc diffusion | CLSI | 158 | 134 |  |  |  | 157 |  | 0 | 6 | (123) |
| 241 | Tshokey Tshokey et al | 2018 | Bhutan | Disc diffusion | WHO | 381 | 324 |  |  |  | 323 |  |  | 7 | (124) |
| 242 | Sidharath D. Thakur et al | 2018 | Canada | MIC base | CLSI | 685 | 12 |  |  |  | 40 |  | 0 | 7 | (125) |
| 244 | Pachara Sirivongrangson et al | 2018 | Thailand | MIC base | CLSI | 3143 | 2904 |  |  |  |  |  |  | 7 | (126) |
| 245 | Mahmuda Naznin et al | 2018 | Bangladesh | Disc diffusion | CLSI | 25 | 17 | 20 |  |  | 20 |  |  | 7 | (127) |
| 249 | Robyn S. Lee et al | 2018 | New Zealand | MIC base | CLSI | 398 | 126 |  |  |  | 106 |  | 0 | 7 | (128) |
| 250 | Sangeeta V. Kulkarni et al | 2018 | India | MIC base | CLSI | 124 | 122 |  |  |  | 57 |  | 0 | 7 | (129) |
| 252 | Shio-Shin Jean et al | 2018 | Taiwan | MIC base | CLSI | 129 | 126 |  |  |  |  |  |  | 7 | (130) |
| 254 | Jennifer Gratrix et al | 2018 | Canada | MIC base | CLSI | 1191 | 362 |  |  |  |  |  |  | 7 | (131) |
| 255 | Jennifer Gratrix et al | 2018 | Canada | MIC base | CLSI | 1898 |  |  |  |  | 301 |  |  | 7 | (131) |
| 256 | Ana Paula Ramalho da Costa-Lourenço et al | 2018 | Brazil | MIC base | CLSI | 116 | 75 |  |  |  | 77 |  |  | 7 | (132) |
| 257 | M. L. Bazzo et al | 2018 | Brazil | MIC base | CLSI | 550 | 306 |  |  |  | 339 |  |  | 7 | (133) |
| 240 | Addisu Gize Yeshanew et al | 2018 | Ethiopia | Disc diffusion | CLSI | 25 | 13 |  |  |  | 25 |  |  | 8 | (134) |
| 268 | Joana Calado et al | 2019 | Pourtugal | MIC base | EUCAST | 30 | 11 |  |  |  | 5 |  | 0 | 5 | (135) |
| 269 | Anna Carannante et al | 2019 | Italy | MIC base | EUCAST | 67 | 41 |  |  |  |  |  |  | 5 | (136) |
| 271 | Fernando Cobo et al | 2019 | Spain | MIC base | EUCAST | 134 | 92 |  |  |  | 74 |  | 0 | 5 | (137) |
| 281 | Santhuri Rambaran et al | 2019 | MC | MIC base | EUCAST | 319 | 223 |  |  |  | 319 |  |  | 5 | (138) |
| 290 | Mary Wandia Kivata et al | 2019 | Kenya | MIC base | EUCAST | 22 | 20 |  |  |  | 16 |  | 0 | 5 | (139) |
| 267 | M. D. Guerrero -Torres et al | 2019 | Spain | MIC base | EUCAST | 16 | 7 |  |  |  | 1 |  | 0 | 6 | (140) |
| 272 | Irya Boiko et al | 2019 | Ukraine | MIC base | EUCAST | 150 | 14 |  | 0 |  | 9 |  | 0 | 6 | (141) |
| 275 | Yijing Yang et al | 2019 | China | MIC base | CLSI | 124 | 123 |  |  |  | 74 |  |  | 6 | (142) |
| 278 | Alain Yéo et al | 2019 | Côte d'Ivoire | MIC base | EUCAST | 212 | 133 |  | 3 |  | 180 |  | 0 | 6 | (143) |
| 283 | Rafael Affini Martins et al | 2019 | Brazil | MIC base | EUCAST | 124 | 19 |  |  |  |  |  |  | 6 | (144) |
| 288 | Jing-Wei Liu et al | 2019 | China | MIC base | EUCAST | 470 |  |  | 0 |  |  |  | 2 | 6 | (145) |
| 292 | Ricardo A. Gianecini et al | 2019 | Argentina | MIC base | EUCAST | 158 |  |  |  |  |  |  | 59 | 6 | (146) |
| 293 | Ricardo A. Gianecini et al | 2019 | Argentina | MIC base | EUCAST | 3478 | 2097 |  |  |  | 1228 |  |  | 6 | (146) |
| 295 | Adam L. Bailey et al | 2019 | USA | MIC base | CLSI | 64 | 4 |  |  |  | 5 |  |  | 6 | (147) |
| 270 | Maartje Visser et al | 2019 | Netherlands | Mixed | EUCAST | 80 | 17 |  |  |  |  |  |  | 7 | (148) |
| 273 | Alexey Kubanov et al | 2019 | Russian | MIC base | CLSI | 444 |  |  |  |  | 111 |  |  | 7 | (149) |
| 277 | Wresti Indriatmi et al | 2019 | Indonesia | Disc diffusion | CLSI | 35 |  |  |  |  | 34 |  |  | 7 | (150) |
| 279 | Jing Yan et al | 2019 | China | MIC base | WHO | 379 | 379 |  |  |  | 303 |  | 0 | 7 | (151) |
| 282 | Xiaolin Qin et al | 2019 | China | MIC base | WHO | 704 | 691 |  |  |  | 603 |  | 0 | 7 | (152) |
| 285 | Emily Mabonga et al | 2019 | Uganda | Disc diffusion | CLSI | 16 | 16 |  | 0 |  | 16 |  |  | 7 | (153) |
| 286 | I Martin et al | 2019 | Canada | MIC base | CLSI | 4538 | 2137 |  |  |  | 2419 |  | 0 | 7 | (154) |
| 296 | Naiki Attram et al | 2019 | Ghana | MIC base | CLSI | 44 | 36 |  |  |  | 44 |  | 0 | 7 | (155) |
| 299 | Daniel Golparian et al | 2020 | Denmark | MIC base | EUCAST | 191 | 2 | 18 | 0 | 0 | 17 | 1 | 0 | 5 | (156) |
| 300 | Daniel Golparian et al | 2020 | Denmark | MIC base | EUCAST | 191 | 122 | 133 | 0 | 0 | 116 | 52 | 0 | 5 | (156) |
| 301 | C. Queirós et al | 2020 | Portugal | Mixed | EUCAST | 425 | 141 |  |  |  | 43 |  |  | 5 | (157) |
| 307 | Pham Thi Lan et al | 2020 | Vietnam | MIC base | EUCAST | 108 | 106 |  |  |  | 89 |  | 0 | 5 | (158) |
| 308 | Pham Thi Lan et al | 2020 | Vietnam | MIC base | EUCAST | 121 | 121 |  |  |  | 96 |  | 0 | 5 | (158) |
| 320 | Miguel Pinto et al | 2020 | Portugal | MIC base | EUCAST | 2551 |  |  |  |  |  |  | 0 | 5 | (159) |
| 322 | Miguel Pinto et al | 2020 | Portugal | MIC base | EUCAST | 2575 | 1109 |  |  |  |  |  |  | 5 | (159) |
| 297 | Irya Boiko et al | 2020 | Ukraine | MIC base | EUCAST | 150 | 17 |  | 0 |  | 9 |  | 0 | 6 | (160) |
| 302 | Urko Ibargoyen García et al | 2020 | Spain | Disc diffusion | CLSI | 731 | 341 |  | 0 |  | 115 |  | 0 | 6 | (161) |
| 303 | Kristian Alfsnes et al | 2020 | Norway | Mixed | EUCAST | 958 | 412 |  |  |  | 316 |  | 0 | 6 | (162) |
| 304 | Katy Town et al | 2020 | England | MIC base | EUCAST | 1277 | 464 |  |  |  |  |  |  | 6 | (163) |
| 331 | Liteboho D. Maduna et al | 2020 | MC | MIC base | EUCAST | 27 | 2 |  |  |  | 20 |  | 0 | 6 | (164) |
| 334 | Daniel Golparian et al | 2020 | Brazil | MIC base | EUCAST | 548 | 300 |  |  |  | 342 |  |  | 6 | (165) |
| 336 | Yuan Dong et al | 2020 | China | MIC base | EUCAST | 366 | 364 |  |  |  | 223 |  | 0 | 6 | (166) |
| 338 | T Crucitti et al | 2020 | Cameroon | Mixed | EUCAST | 396 | 255 |  |  |  |  |  |  | 6 | (167) |
| 339 | T Crucitti et al | 2020 | Cameroon | Mixed | EUCAST | 411 |  |  |  |  | 240 |  |  | 6 | (167) |
| 342 | T Crucitti et al | 2020 | Cameroon | Mixed | EUCAST | 410 |  |  |  |  |  |  | 8 | 6 | (167) |
| 306 | Paula Salmerón et al | 2020 | Spain | MIC base | CLSI | 2036 | 1044 |  |  |  |  |  | 0 | 7 | (168) |
| 316 | Feng Wang et al | 2020 | China | MIC base | WHO | 1282 | 1249 |  |  |  |  |  | 0 | 7 | (169) |
| 324 | Carrie Nacht et al | 2020 | Kenya | Disc diffusion | CLSI | 35 |  |  | 18 |  |  |  |  | 7 | (170) |
| 325 | Carrie Nacht et al | 2020 | Kenya | Disc diffusion | CLSI | 34 | 34 |  |  |  | 34 |  |  | 7 | (170) |
| 330 | Tatum D. Mortimer et al | 2020 | USA | MIC base | CLSI | 889 | 216 |  |  |  |  |  |  | 7 | (171) |
| 333 | Ranmini Kularatne et al | 2020 | MC | MIC base | CLSI | 272 |  |  | 26 |  |  |  |  | 7 | (172) |
| 335 | Surafel Fentaw et al | 2020 | Ethiopia | Mixed | CLSI | 361 | 217 |  |  |  |  |  | 11 | 7 | (173) |
| 343 | Paul C. Adamson et al | 2020 | Vietnam | Disc diffusion | CLSI | 409 | 402 |  |  |  | 337 |  |  | 7 | (174) |
| 345 | Paul C. Adamson et al | 2020 | Vietnam | Disc diffusion | CLSI | 408 |  |  |  |  |  |  | 0 | 7 | (174) |
| 346 | Zhou Zheng et al | 2020 | China | MIC base | CLSI | 55 | 50 |  |  |  | 19 |  | 0 | 7 | (175) |
| 319 | Addisu Sahile et al | 2020 | Ethiopia | Disc diffusion | CLSI | 31 | 4 |  |  |  | 17 |  | 0 | 8 | (176) |
| 350 | Magnus Unemo et al | 2021 | MC | MIC base | EUCAST | 1209 | 603 |  |  |  |  |  |  | 5 | (177) |
| 357 | Jing Yan et al | 2021 | China | MIC base | EUCAST | 70 | 70 |  |  |  | 41 |  | 0 | 5 | (178) |
| 358 | Masatoshi Tanaka et al | 2021 | Japan | MIC base | CLSI | 17 | 2 |  |  |  | 9 |  | 0 | 5 | (179) |
| 359 | Masatoshi Tanaka et al | 2021 | Japan | MIC base | CLSI | 33 | 29 |  |  |  | 10 |  | 0 | 5 | (179) |
| 363 | Manuel C. Jamoralin et al | 2021 | Philippines | MIC base | CLSI | 21 | 19 |  |  |  | 14 |  |  | 5 | (180) |
| 348 | Jolinda de Korne-Elenbaas et al | 2021 | Netherlands | MIC base | EUCAST | 318 | 172 |  |  |  |  |  |  | 6 | (181) |
| 349 | Aliaksandra Aniskevich et al | 2021 | Belarus | MIC base | EUCAST | 522 | 129 |  | 0 |  | 145 |  | 0 | 6 | (182) |
| 352 | Paula Salmero´n et al | 2021 | Spain | MIC base | EUCAST | 2416 | 1358 |  |  |  |  |  |  | 6 | (183) |
| 361 | Saliya Karymbaeva et al | 2021 | Kyrgyzstan | MIC base | EUCAST | 156 | 138 |  | 1 | 4 | 41 |  | 0 | 6 | (184) |
| 362 | Francis Kakooza et al | 2021 | Uganda | MIC base | CLSI | 458 | 456 |  | 0 |  |  |  |  | 6 | (185) |
| 368 | Susanne Jacobsson et al | 2021 | MC | MIC base | EUCAST | 15792 | 8164 |  |  |  |  |  |  | 6 | (186) |
| 356 | Bingming Zhu et al | 2021 | China | MIC base | WHO | 304 | 264 |  |  |  |  |  |  | 7 | (187) |
| 370 | Wenjing Le et al | 2021 | China | MIC base | CLSI | 986 | 986 |  |  |  | 832 |  | 0 | 7 | (188) |

1. Fekete T, Serfass D, Lafredo S, Cundy K. Susceptibility to cephalosporins of penicillin-susceptible and penicillin-resistant strains of Neisseria gonorrhoeae from Philadelphia. Antimicrobial agents and chemotherapy. 1989;33(2):164-6.

2. Loo VG, Simor AE, Jaeger R, Low DE. Survey of Neisseria gonorrhoeae antimicrobial susceptibility in Ontario. Canadian Journal of Infectious Diseases. 1990;1(4):136-8.

3. Fekete T, Woodwell J, Cundy K. Susceptibility of Neisseria gonorrhoeae to cefpodoxime: determination of MICs and disk diffusion zone diameters. Antimicrobial agents and chemotherapy. 1991;35(3):497-9.

4. Putnam S, Lavin B, Stone J, Oldfield 3rd E, Hooper D. Evaluation of the standardized disk diffusion and agar dilution antibiotic susceptibility test methods by using strains of Neisseria gonorrhoeae from the United States and Southeast Asia. Journal of Clinical Microbiology. 1992;30(4):974-80.

5. Rice RJ, Knapp JS. Susceptibility of Neisseria gonorrhoeae associated with pelvic inflammatory disease to cefoxitin, ceftriaxone, clindamycin, gentamicin, doxycycline, azithromycin, and other antimicrobial agents. Antimicrobial agents and chemotherapy. 1994;38(7):1688-91.

6. Rice RJ, Knapp JS. Antimicrobial susceptibilities of Neisseria gonorrhoeae strains representing five distinct resistance phenotypes. Antimicrobial agents and chemotherapy. 1994;38(1):155-8.

7. Joesoef M, Knapp J, Idajadi A, Linnan M, Barakbah Y, Kamboji A, et al. Antimicrobial susceptibilities of Neisseria gonorrhoeae strains isolated in Surabaya, Indonesia. Antimicrobial agents and chemotherapy. 1994;38(11):2530-3.

8. West B, Changalucha J, Grosskurth H, Mayaud P, Gabone R, Ka-Gina G, et al. Antimicrobial susceptibility, auxotype and plasmid content of Neisseria gonorrhoeae in northern Tanzania: emergence of high level plasmid mediated tetracycline resistance. Sexually Transmitted Infections. 1995;71(1):9-12.

9. Knapp JS, Brathwaite AR, Hinds A, Duncan W, Rice RJ. Plasmid-mediated antimicrobial resistance in Neisseria gonorrhoeae in Kingston, Jamaica: 1990-1991. Sexually transmitted diseases. 1995:155-9.

10. Cummings MC, Covino JM, Smith BL, Ratiu ES, Draft K, McCORMACK WM. Susceptibility of Isolates of Neisseria gonorrhoeae to Penicillin and Tetracycline in Brooklyn, 1988-1992. Sexually transmitted diseases. 1995:110-3.

11. Kam KM, WONG PW, Cheung MM, HO NKY, Lo KK. Quinolone-ResistantNeisseria gonorrhoeaein Hong Kong. Sexually transmitted diseases. 1996;23(2):103-8.

12. Nissinen A, JÄRVINEN H, Liimatainen O, Jahkola M, Huovinen P, Resistance FSGfA. Antimicrobial resistance in Neisseria gonorrhoeae in Finland, 1976 to 1995. Sexually transmitted diseases. 1997:576-81.

13. Adegbola R, Sabally S, Corrah T, West B, Mabey D. Increasing prevalence of penicillinase‐producing Neisseria gonorrhoeae and the emergence of high‐level, plasmid‐mediated tetracycline resistance among gonococcal isolates in The Gambia. Tropical Medicine & International Health. 1997;2(5):428-32.

14. Knapp JS, Wongba C, Limpakarnjanarat K, Young NL, Parekh MC, Neal SW, et al. Antimicrobial susceptibilities of strains of Neisseria gonorrhoeae in Bangkok, Thailand: 1994-1995. Sexually transmitted diseases. 1997:142-8.

15. Guyot A, Jarrett B, Sanvee L, Dore D. Antimicrobial resistance of Neisseria gonorrhoeae in Liberia. Transactions of the Royal Society of Tropical Medicine and Hygiene. 1998;92(6):670-4.

16. Bhuiyan BU, Rahman M, Miah MRA, Nahar S, Islam N, Ahmed M, et al. Antimicrobial susceptibilities and plasmid contents of Neisseria gonorrhoeae isolates from commercial sex workers in Dhaka, Bangladesh: emergence of high-level resistance to ciprofloxacin. Journal of clinical microbiology. 1999;37(4):1130-6.

17. Ray K, Bala M, Kumar J, Misra R. Trend of antimicrobial resistance in Neisseria gonorrhoeae at New Delhi, India. International journal of STD & AIDS. 2000;11(2):115-8.

18. Wenling C, Xibao Z, MINCHANG W, PING L, DEBIAO W, JINLAN Y. Analysis of the antibiotic sensitivity of Neisseria gonorrhoeae in Guangzhou, Peoples Republic of China. Sexually transmitted diseases. 2000:480-2.

19. Dillon J-AR, Rubabaza J-PA, Benzaken AS, Sardinha JCG, Li H, Bandeira MGC, et al. Reduced susceptibility to azithromycin and high percentages of penicillin and tetracycline resistance in Neisseria gonorrhoeae isolates from Manaus, Brazil, 1998. Sexually transmitted diseases. 2001:521-6.

20. Ye S, Su X, Wang Q, Yin Y, Dai X, Sun H. Surveillance of antibiotic resistance of Neisseria gonorrhoeae isolates in China, 1993–1998. Sexually transmitted diseases. 2002;29(4):242-5.

21. Ng L-K, Sawatzky P, Martin IE, Booth S. Characterization of ciprofloxacin resistance in Neisseria gonorrhoeae isolates in Canada. Sexually transmitted diseases. 2002:780-8.

22. Llanes R, Sosa J, Guzman D, Llop A, Valdés EA, Martínez I, et al. Antimicrobial susceptibility of Neisseria gonorrhoeae in Cuba (1995-1999): implications for treatment of gonorrhea. Sexually transmitted diseases. 2003:10-4.

23. Trees DL, Sirivongrangson P, Schultz AJ, Buatiang A, Neal SW, Knapp JS, et al. Multiclonal increase in ciprofloxacin-resistant Neisseria gonorrhoeae, Thailand, 1998-1999. Sexually transmitted diseases. 2002:668-73.

24. Rahman M, Sultan Z, Monira S, Alam A, Nessa K, Islam S, et al. Antimicrobial susceptibility of Neisseria gonorrhoeae isolated in Bangladesh (1997 to 1999): rapid shift to fluoroquinolone resistance. Journal of Clinical Microbiology. 2002;40(6):2037-40.

25. Ieven M, Van Looveren M, Sudigdoadi S, Rosana Y, Goossens W, Lammens C, et al. Antimicrobial susceptibilities of Neisseria gonorrhoeae strains isolated in Java, Indonesia. Sexually transmitted diseases. 2003:25-9.

26. Bhalla P, Vidhani S, Reddy B, Chowdhry S, Mathur M. Rising quinolone resistance in Neisseria gonorrhoeae isolates from New Delhi. Indian Journal of Medical Research. 2002;115:113.

27. Dan M, Poch F, Sheinberg B. High prevalence of high-level ciprofloxacin resistance in Neisseria gonorrhoeae in Tel Aviv, Israel: correlation with response to therapy. Antimicrobial agents and chemotherapy. 2002;46(6):1671-3.

28. Sosa J, Ramirez-Arcos S, Ruben M, Li H, Llanes R, Llop A, et al. High Percentages of Resistance to Tetracycline and Penicillin and Reduced Susceptibility to Azithromycin Characterize the Majority of Strain Types of Isolates in Cuba, 1995-1998. Sexually transmitted diseases. 2003:443-8.

29. Ehinmidu J, Bolaji R, Adegboye E. Isolation and antibiotic susceptibility profile of Neisseria gonorrhoeae isolated from urine samples in Zara, Northern Nigeria. Journal of Phytomedicine and Therapeutics. 2004;9.

30. Shigemura K, Tanaka K, Arakawa S, Fujisawa M, Shirakawa T, Massi N, et al. Presence of a mutation in ponA1 of Neisseria gonorrhoeae in numerous clinical samples resistant to various β-lactams and other, structurally unrelated, antimicrobials. Journal of infection and chemotherapy. 2005;11(5):226-30.

31. Yoo J, Yoo C, Cho Y, Park H, Oh H-B, Seong WK. Antimicrobial resistance patterns (1999-2002) and characterization of ciprofloxacin-resistant Neisseria gonorrhoeae in Korea. Sexually transmitted diseases. 2004:305-10.

32. Aydın D, Küçükbasmacı Ö, Gönüllü N, Aktaş Z. Susceptibilities of Neisseria gonorrhoeae and Ureaplasma urealyticum isolates from male patients with urethritis to several antibiotics including telithromycin. Chemotherapy. 2005;51(2-3):89-92.

33. Yang Y, Liao M, Gu W-M, Bell K, Wu L, Eng NF, et al. Antimicrobial susceptibility and molecular determinants of quinolone resistance in Neisseria gonorrhoeae isolates from Shanghai. Journal of Antimicrobial Chemotherapy. 2006;58(4):868-72.

34. Enders M, Turnwald-Maschler A, Regnath T. Antimicrobial resistance of Neisseria gonorrhoeae isolates from the Stuttgart and Heidelberg areas of southern Germany. European Journal of Clinical Microbiology and Infectious Diseases. 2006;25(5):318-22.

35. Sethi S, Sharma D, Mehta S, Singh B, Smriti M, Kumar B, et al. Emergence of ciprofloxacin resistant Neisseria gonorrhoeae in north India. Indian J Med Res. 2006;123(5):707-10.

36. Donegan EA, Wirawan DN, Muliawan P, Schachter J, Moncada J, Parekh M, et al. Fluoroquinolone-resistant Neisseria gonorrhoeae in Bali, Indonesia: 2004. Sexually transmitted diseases. 2006;33(10):625-9.

37. Wang B, Xu J-s, Wang C-x, Mi Z-h, Pu Y-p, Hui M, et al. Antimicrobial susceptibility of Neisseria gonorrhoeae isolated in Jiangsu Province, China, with a focus on fluoroquinolone resistance. Journal of medical microbiology. 2006;55(9):1251-5.

38. Khaki P, Bhalla P, Sharma P, Chawla R, Bhalla K. Epidemilogical analysis of Neisseria gonorrhoeae isolates by antimicrobial susceptibility testing, auxotyping and serotyping. Indian Journal of Medical Microbiology. 2007;25(3):225-9.

39. Su X, Jiang F, Dai X, Sun H, Ye S. Surveillance of antimicrobial susceptibilities in Neisseria gonorrhoeae in Nanjing, China, 1999-2006. Sexually transmitted diseases. 2007:995-9.

40. Vorobieva V, Firsova N, Ababkova T, Leniv I, Haldorsen BC, Unemo M, et al. Antibiotic susceptibility of Neisseriagonorrhoeae in Arkhangelsk, Russia. Sexually transmitted infections. 2007;83(2):133-5.

41. Hovhannisyan G, von Schoen-Angerer T, Babayan K, Fenichiu O, Gaboulaud V. Antimicrobial susceptibility of Neisseria gonorrheae strains in three regions of Armenia. Sexually transmitted diseases. 2007;34(9):686-8.

42. Bala M, Ray K, Gupta S. Antimicrobial resistance pattern of Neisseria gonorrhoeae isolates from peripheral health centres and STD clinic attendees of a tertiary care centre in India. International journal of STD & AIDS. 2008;19(6):378-80.

43. Lewis DA, Scott L, Slabbert M, Mhlongo S, van Zijl A, Sello M, et al. Escalation in the relative prevalence of ciprofloxacin-resistant gonorrhoea among men with urethral discharge in two South African cities: association with HIV seropositivity. Sexually transmitted infections. 2008;84(5):352-5.

44. Cao V, Ratsima E, Van Tri D, Bercion R, Fonkoua M-C, Richard V, et al. Antimicrobial susceptibility of Neisseria gonorrhoeae strains isolated in 2004-2006 in Bangui, Central African Republic; Yaoundé, Cameroon; Antananarivo, Madagascar; and Ho Chi Minh Ville and Nha Trang, Vietnam. Sexually transmitted diseases. 2008:941-5.

45. Starnino S, Suligoi B, Regine V, Bilek N, Stefanelli P, Dal Conte I, et al. Phenotypic and genotypic characterization of Neisseria gonorrhoeae in parts of Italy: detection of a multiresistant cluster circulating in a heterosexual network. 2008; 14: 949–954. 2008.

46. Lis‐Tønder J, Cybulski Z. Antimicrobial susceptibility and biochemical patterns of Neisseria gonorrhoeae strains in Vejle area, Denmark. Journal of the European Academy of Dermatology and Venereology. 2009;23(10):1193-6.

47. Morris SR, Moore DF, Hannah PB, Wang SA, Wolfe J, Trees DL, et al. Strain typing and antimicrobial resistance of fluoroquinolone-resistant Neisseria gonorrhoeae causing a California infection outbreak. Journal of Clinical Microbiology. 2009;47(9):2944-9.

48. Apalata T, Zimba TF, Sturm WA, Moodley P. Antimicrobial susceptibility profile of Neisseria gonorrhoeae isolated from patients attending a STD facility in Maputo, Mozambique. Sexually transmitted diseases. 2009;36(6):341-3.

49. Brown LB, Krysiak R, Kamanga G, Mapanje C, Kanyamula H, Banda B, et al. Neisseria gonorrhoeae antimicrobial susceptibility in Lilongwe, Malawi, 2007. Sexually transmitted diseases. 2010;37(3):169-72.

50. Ahmed MU, Chawdhury FAH, Hossain M, Sultan SZ, Alam M, Salahuddin G, et al. Monitoring antimicrobial susceptibility of Neisseria gonorrhoeae isolated from Bangladesh during 1997-2006: emergence and pattern of drug-resistant isolates. Journal of health, population, and nutrition. 2010;28(5):443.

51. Dan M, Mor Z, Gottliev S, Sheinberg B, Shohat T. Trends in antimicrobial susceptibility of Neisseria gonorrhoeae in Israel, 2002 to 2007, with special reference to fluoroquinolone resistance. Sexually transmitted diseases. 2010;37(7):451-3.

52. Kubanova A, Frigo N, Kubanov A, Sidorenko S, Lesnaya I, Polevshikova S, et al. The Russian gonococcal antimicrobial susceptibility programme (RU-GASP)–national resistance prevalence in 2007 and 2008, and trends during 2005-2008. Eurosurveillance. 2010;15(14):19533.

53. Tanaka M, Koga Y, Nakayama H, Kanayama A, Kobayashi I, Saika T, et al. Antibiotic-resistant phenotypes and genotypes of Neisseria gonorrhoeae isolates in Japan: identification of strain clusters with multidrug-resistant phenotypes. Sexually transmitted diseases. 2011;38(9):871-5.

54. Glazkova S, Golparian D, Titov L, Pankratova N, Suhabokava N, Shimanskaya I, et al. Antimicrobial susceptibility/resistance and molecular epidemiological characteristics of Neisseria gonorrhoeae in 2009 in Belarus. Apmis. 2011;119(8):537-42.

55. Allen VG, Farrell DJ, Rebbapragada A, Tan J, Tijet N, Perusini SJ, et al. Molecular analysis of antimicrobial resistance mechanisms in Neisseria gonorrhoeae isolates from Ontario, Canada. Antimicrobial agents and chemotherapy. 2011;55(2):703-12.

56. Mehta SD, Maclean I, Ndinya-Achola JO, Moses S, Martin I, Ronald A, et al. Emergence of quinolone resistance and cephalosporin MIC creep in Neisseria gonorrhoeae isolates from a cohort of young men in Kisumu, Kenya, 2002 to 2009. Antimicrobial agents and chemotherapy. 2011;55(8):3882-8.

57. Olsen B, Månsson F, Camara C, Monteiro M, Biai A, Alves A, et al. Phenotypic and genetic characterisation of bacterial sexually transmitted infections in Bissau, Guinea-Bissau, West Africa: a prospective cohort study. BMJ open. 2012;2(2):e000636.

58. Martin I, Sawatzky P, Allen V, Hoang L, Lefebvre B, Mina N, et al. Emergence and characterization of Neisseria gonorrhoeae isolates with decreased susceptibilities to ceftriaxone and cefixime in Canada: 2001–2010. Sexually transmitted diseases. 2012:316-23.

59. Hjelmevoll S, Golparian D, Dedi L, Skutlaberg D, Haarr E, Christensen A, et al. Phenotypic and genotypic properties of Neisseria gonorrhoeae isolates in Norway in 2009: antimicrobial resistance warrants an immediate change in national management guidelines. European journal of clinical microbiology & infectious diseases. 2012;31(6):1181-6.

60. Lo JYC, Ho KM, Lo ACT. Surveillance of gonococcal antimicrobial susceptibility resulting in early detection of emerging resistance. Journal of antimicrobial chemotherapy. 2012;67(6):1422-6.

61. Hamasuna R, Yasuda M, Ishikawa K, Uehara S, Takahashi S, Hayami H, et al. Nationwide surveillance of the antimicrobial susceptibility of Neisseria gonorrhoeae from male urethritis in Japan. Journal of Infection and Chemotherapy. 2013;19(4):571-8.

62. Kirkcaldy RD, Zaidi A, Hook III EW, Holmes KK, Soge O, del Rio C, et al. Neisseria gonorrhoeae antimicrobial resistance among men who have sex with men and men who have sex exclusively with women: the Gonococcal Isolate Surveillance Project, 2005–2010. Annals of internal medicine. 2013;158(5_Part_1):321-8.

63. Sood S, Mahajan N, Verma R, Kar H, Sharma V. Emergence of decreased susceptibility to extended-spectrum cephalosporins in Neisseria gonorrhoeae in India. The National medical journal of India. 2013;26(1):26-8.

64. Shimuta K, Unemo M, Nakayama S-i, Morita-Ishihara T, Dorin M, Kawahata T, et al. Antimicrobial resistance and molecular typing of Neisseria gonorrhoeae isolates in Kyoto and Osaka, Japan, 2010 to 2012: intensified surveillance after identification of the first strain (H041) with high-level ceftriaxone resistance. Antimicrobial agents and chemotherapy. 2013;57(11):5225-32.

65. Sethi S, Golparian D, Bala M, Dorji D, Ibrahim M, Jabeen K, et al. Antimicrobial susceptibility and genetic characteristics of Neisseria gonorrhoeae isolates from India, Pakistan and Bhutan in 2007–2011. BMC infectious diseases. 2013;13(1):1-8.

66. Dillon J-AR, Trecker MA, Thakur SD. Two decades of the gonococcal antimicrobial surveillance program in South America and the Caribbean: challenges and opportunities. Sexually transmitted infections. 2013;89(Suppl 4):iv36-iv41.

67. Olsen B, Lan PT, Golparian D, Johansson E, Khang TH, Unemo M. Antimicrobial susceptibility and genetic characteristics of Neisseria gonorrhoeae isolates from Vietnam, 2011. BMC infectious diseases. 2013;13(1):1-8.

68. Martin I, Sawatzky P, Liu G, Allen V, Lefebvre B, Hoang L, et al. Antimicrobial susceptibilities and distribution of sequence types of Neisseria gonorrhoeae isolates in Canada: 2010. Canadian journal of microbiology. 2013;59(10):671-8.

69. Costa LMB, Pedroso ERP, Vieira Neto V, Souza VCP, Teixeira MJB. Antimicrobial susceptibility of Neisseria gonorrhoeae isolates from patients attending a public referral center for sexually transmitted diseases in Belo Horizonte, State of Minas Gerais, Brazil. Revista da Sociedade Brasileira de Medicina Tropical. 2013;46:304-9.

70. Hançali A, Ndowa F, Bellaji B, Bennani A, Kettani A, Charof R, et al. Antimicrobial resistance monitoring in Neisseria gonorrhoeae and strategic use of funds from the Global Fund to set up a systematic Moroccan gonococcal antimicrobial surveillance programme. Sexually Transmitted Infections. 2013;89(Suppl 4):iv24-iv7.

71. La Ruche G, Goubard A, Bercot B, Cambau E, Semaille C, Sednaoui P. Gonococcal infections and emergence of gonococcal decreased susceptibility to cephalosporins in France, 2001 to 2012. Eurosurveillance. 2014;19(34):20885.

72. Nemes-Nikodém É, Brunner A, Pintér D, Mihalik N, Lengyel G, Marschalkó M, et al. Antimicrobial susceptibility and genotyping analysis of Hungarian Neisseria gonorrhoeae strains in 2013. Acta Microbiologica et Immunologica Hungarica. 2014;61(4):435-45.

73. Li S, Su X-H, Le W-J, Jiang F-X, Wang B-X, Rice PA. Antimicrobial susceptibility of Neisseria gonorrhoeaeisolates from symptomatic men attending the Nanjing sexually transmitted diseases clinic (2011–2012): genetic characteristics of isolates with reduced sensitivity to ceftriaxone. BMC infectious diseases. 2014;14(1):1-10.

74. Gu W-M, Chen Y, Yang Y, Wu L, Hu W-Z, Jin Y-L. Twenty-five-year changing pattern of gonococcal antimicrobial susceptibility in Shanghai: surveillance and its impact on treatment guidelines. BMC infectious diseases. 2014;14(1):1-8.

75. Jeverica S, Golparian D, Matičič M, Potočnik M, Mlakar B, Unemo M. Phenotypic and molecular characterization of Neisseria gonorrhoeae isolates from Slovenia, 2006–12: rise and fall of the multidrug-resistant NG-MAST genogroup 1407 clone? Journal of Antimicrobial Chemotherapy. 2014;69(6):1517-25.

76. Horn NN, Kresken M, Körber-Irrgang B, Göttig S, Wichelhaus C, Wichelhaus TA. Antimicrobial susceptibility and molecular epidemiology of Neisseria gonorrhoeae in Germany. International Journal of Medical Microbiology. 2014;304(5-6):586-91.

77. Golparian D, Brilene T, Laaring Y, Viktorova E, Johansson E, Domeika M, et al. First antimicrobial resistance data and genetic characteristics of Neisseria gonorrhoeae isolates from Estonia, 2009–2013. New microbes and new infections. 2014;2(5):150-3.

78. Mlynarczyk-Bonikowska B, Serwin AB, Golparian D, Walter de Walthoffen S, Majewski S, Koper M, et al. Antimicrobial susceptibility/resistance and genetic characteristics of Neisseria gonorrhoeaeisolates from Poland, 2010-2012. BMC Infectious Diseases. 2014;14(1):1-7.

79. Chen S-C, Yin Y-P, Dai X-Q, Unemo M, Chen X-S. Antimicrobial resistance, genetic resistance determinants for ceftriaxone and molecular epidemiology of Neisseria gonorrhoeae isolates in Nanjing, China. Journal of Antimicrobial Chemotherapy. 2014;69(11):2959-65.

80. Brunner A, Nemes-Nikodem E, Mihalik N, Marschalko M, Karpati S, Ostorhazi E. Incidence and antimicrobial susceptibility of Neisseria gonorrhoeae isolates from patients attending the national Neisseria gonorrhoeaereference laboratory of Hungary. BMC Infectious Diseases. 2014;14(1):1-8.

81. Zhu B-y, Yu R-x, Yin Y, Chen X, Li W, Dai X-q, et al. Surveillance of antimicrobial susceptibilities of Neisseria gonorrhoeae in Nanning, China, 2000 to 2012. Sexually Transmitted Diseases. 2014;41(8):501-6.

82. Zheng H, Yang B, Wu X, Huang J, Zeng W, Xue Y, et al. Antibiotic susceptibility of Neisseria gonorrhoeae isolates from Guangzhou, China, during 2002–2011. Japanese journal of infectious diseases. 2014;67(4):288-91.

83. Vandepitte J, Hughes P, Matovu G, Bukenya J, Grosskurth H, Lewis DA. High prevalence of ciprofloxacin-resistant gonorrhea among female sex workers in Kampala, Uganda (2008—2009). Sexually transmitted diseases. 2014;41(4):233-7.

84. Takuva S, Mugurungi O, Mutsvangwa J, Machiha A, Mupambo AC, Maseko V, et al. Etiology and antimicrobial susceptibility of pathogens responsible for urethral discharge among men in Harare, Zimbabwe. Sexually transmitted diseases. 2014;41(12):713-7.

85. Peterson S, Martin I, Demczuk W, Bharat A, Hoang L, Wylie J, et al. Molecular assay for detection of ciprofloxacin resistance in Neisseria gonorrhoeae isolates from cultures and clinical nucleic acid amplification test specimens. Journal of clinical microbiology. 2015;53(11):3606-8.

86. Cole MJ, Spiteri G, Jacobsson S, Pitt R, Grigorjev V, Unemo M. Is the tide turning again for cephalosporin resistance in Neisseria gonorrhoeae in Europe? Results from the 2013 European surveillance. BMC infectious diseases. 2015;15(1):1-8.

87. Cobo F, Cabezas-Fernández MT, Cabeza-Barrera MI. Antimicrobial susceptibility and typing of Neisseria gonorrhoeae strains from Southern Spain, 2012–2014. Enfermedades Infecciosas y Microbiología Clínica. 2016;34(1):3-7.

88. Lebedzeu F, Golparian D, Titov L, Pankratava N, Glazkova S, Shimanskaya I, et al. Antimicrobial susceptibility/resistance and NG-MAST characterisation of Neisseria gonorrhoeae in Belarus, Eastern Europe, 2010–2013. BMC infectious diseases. 2015;15(1):1-7.

89. Tanaka M, Furuya R, Irie S, Kanayama A, Kobayashi I. High prevalence of azithromycin-resistant Neisseria gonorrhoeae isolates with a multidrug resistance phenotype in Fukuoka, Japan. Sexually Transmitted Diseases. 2015;42(6):337-41.

90. Lee H, Unemo M, Kim HJ, Seo Y, Lee K, Chong Y. Emergence of decreased susceptibility and resistance to extended-spectrum cephalosporins in Neisseria gonorrhoeae in Korea. Journal of Antimicrobial Chemotherapy. 2015;70(9):2536-42.

91. Hamasuna R, Yasuda M, Ishikawa K, Uehara S, Hayami H, Takahashi S, et al. The second nationwide surveillance of the antimicrobial susceptibility of Neisseria gonorrhoeae from male urethritis in Japan, 2012–2013. Journal of Infection and Chemotherapy. 2015;21(5):340-5.

92. Bharara T, Bhalla P, Rawat D, Garg V, Sardana K, Chakravarti A. Rising trend of antimicrobial resistance among Neisseria gonorrhoeae isolates and the emergence of N. gonorrhoeae isolate with decreased susceptibility to ceftriaxone. Indian journal of medical microbiology. 2015;33(1):39-42.

93. Kubanov A, Vorobyev D, Chestkov A, Leinsoo A, Shaskolskiy B, Dementieva E, et al. Molecular epidemiology of drug-resistant Neisseria gonorrhoeae in Russia (Current Status, 2015). BMC infectious diseases. 2016;16(1):1-10.

94. Khanam R, Ahmed D, Rahman M, Alam M, Amin M, Khan SI, et al. Antimicrobial susceptibility of Neisseria gonorrhoeae in Bangladesh (2014 update). Antimicrobial agents and chemotherapy. 2016;60(7):4418-9.

95. Azizmohammadi S, Azizmohammadi S. Antimicrobial susceptibility pattern of Neisseria gonorrhoeae isolated from fertile and infertile women. Tropical Journal of Pharmaceutical Research. 2016;15(12):2653-7.

96. Carannante A, Vacca P, Ghisetti V, Latino MA, Cusini M, Matteelli A, et al. Genetic resistance determinants for cefixime and molecular analysis of gonococci isolated in Italy. Microbial Drug Resistance. 2017;23(2):247-52.

97. de Vega IF, Baliu-Piqué C, Mestres JB, Gómez AV, Vallés X, Gibert MA. Risk factors for antimicrobial-resistant Neisseria gonorrhoeae and characteristics of patients infected with gonorrhea. Enfermedades Infecciosas y Microbiología Clínica. 2018;36(3):165-8.

98. Brunner A, Nemes-Nikodem E, Jeney C, Szabo D, Marschalko M, Karpati S, et al. Emerging azithromycin-resistance among the Neisseria gonorrhoeae strains isolated in Hungary. Annals of clinical microbiology and antimicrobials. 2016;15(1):1-6.

99. Serra-Pladevall J, Barberá M, Rodriguez S, Bartolomé-Comas R, Roig G, Juvé R, et al. Neisseria gonorrhoeae antimicrobial susceptibility in Barcelona: penA, ponA, mtrR, and porB mutations and NG-MAST sequence types associated with decreased susceptibility to cephalosporins. European Journal of Clinical Microbiology & Infectious Diseases. 2016;35(9):1549-56.

100. Tribuddharat C, Pongpech P, Charoenwatanachokchai A, Lokpichart S, Srifuengfung S, Sonprasert S. Gonococcal antimicrobial susceptibility and prevalence of blaTEM-1, blaTEM-135 genes in Thailand. Japanese journal of infectious diseases. 2016:JJID. 2016.209.

101. Regnath T, Mertes T, Ignatius R. Antimicrobial resistance of Neisseria gonorrhoeae isolates in south-west Germany, 2004 to 2015: increasing minimal inhibitory concentrations of tetracycline but no resistance to third-generation cephalosporins. Eurosurveillance. 2016;21(36):30335.

102. Jabeen K, Bhawan Mal P, Khan E, Chandio S, Jacobsson S, Unemo M. Antimicrobial resistance and Neisseria gonorrhoeae multiantigen sequence typing (NG-MAST) genotypes in N. gonorrhoeae during 2012–2014 in Karachi, Pakistan. BMC infectious diseases. 2016;16(1):1-6.

103. Lin Y-H, Huang H-C, Hung C-H, Lu Y-C, Jiang B-J, Chou Y-W, et al. Increased incidences of multidrug-resistant gonorrhea in Taiwanese men: Experiences from a single institute. Urological Science. 2016;27(2):86-90.

104. Cheng C-W, Li L-H, Su C-Y, Li S-Y, Yen M-Y. Changes in the six most common sequence types of Neisseria gonorrhoeae, including ST4378, identified by surveillance of antimicrobial resistance in northern Taiwan from 2006 to 2013. Journal of Microbiology, Immunology and Infection. 2016;49(5):708-16.

105. Yu R-x, Yin Y, Dai X-q, Chen S-c, Han Y, Zheng B-j, et al. Antimicrobial Susceptibility Evaluation and Multiple-Locus Variable Number Tandem Repeat Analysis of Neisseria gonorrhoeae Isolates in China in 2012. Sexually Transmitted Diseases. 2017;44(4):197-201.

106. Lagacé-Wiens P, Adam H, Laing N, Baxter M, Martin I, Mulvey M, et al. Antimicrobial susceptibility of clinical isolates of Neisseria gonorrhoeae to alternative antimicrobials with therapeutic potential. Journal of Antimicrobial Chemotherapy. 2017;72(8):2273-7.

107. Serra-Pladevall J, Barberá M, Callarisa A, Bartolomé-Comas R, Andreu A. Differences in Neisseria gonorrhoeae population structure and antimicrobial resistance pattern between men who have sex with men and heterosexuals. Epidemiology & Infection. 2017;145(2):379-85.

108. Li W, Zhu B-y, Qin S-q, Yang M-c, Liang M, He S, et al. Surveillance of antimicrobial susceptibilities of Neisseria gonorrhoeae from 2013 to 2015 in Guangxi Province, China. Japanese Journal of Infectious Diseases. 2017:JJID. 2017.169.

109. Gianecini R, de las Mercedes Romero M, Oviedo C, Vacchino M, Galarza P. Emergence and spread of Neisseria gonorrhoeae isolates with decreased susceptibility to extended-spectrum cephalosporins in Argentina, 2009 to 2013. Sexually Transmitted Diseases. 2017;44(6):351-5.

110. Araneta CMP, Juayang AC, Lim JPT, Quilop EMG, Casaysay NJG, Tamesis GML, et al. Antibiotic susceptibility monitoring of Neisseria gonorrhoeae in bacolod city, philippines. Tropical medicine and infectious disease. 2017;2(3):45.

111. Latif AS, Gwanzura L, Machiha A, Ndowa F, Tarupiwa A, Gudza-Mugabe M, et al. Antimicrobial susceptibility in Neisseria gonorrhoeae isolates from five sentinel surveillance sites in Zimbabwe, 2015–2016. Sexually Transmitted Infections. 2018;94(1):62-6.

112. Peng T, Lin H, Liu Q, Cao W, Ding H, Chen J, et al. Ceftriaxone susceptibility and molecular characteristics of Neisseria gonorrhoeae isolates in Changsha, China. Journal of Infection and Chemotherapy. 2017;23(6):385-9.

113. Sood S, Agarwal M, Bala M, Mahajan N, Singh R, Kapil A, et al. Exploring quinolone resistance-determining region in Neisseria gonorrhoeae isolates from across India. The Indian Journal of Medical Research. 2017;146(Suppl 1):S64.

114. Jiang F-X, Lan Q, Le W-J, Su X-H. Antimicrobial susceptibility of Neisseria gonorrhoeae isolates from Hefei (2014–2015): genetic characteristics of antimicrobial resistance. BMC infectious diseases. 2017;17(1):1-6.

115. Hofstraat SH, Götz HM, van Dam AP, van der Sande MA, van Benthem BH. Trends and determinants of antimicrobial susceptibility of Neisseria gonorrhoeae in the Netherlands, 2007 to 2015. Eurosurveillance. 2018;23(36):1700565.

116. Buder S, Dudareva S, Jansen K, Loenenbach A, Nikisins S, Sailer A, et al. Antimicrobial resistance of Neisseria gonorrhoeae in Germany: low levels of cephalosporin resistance, but high azithromycin resistance. BMC Infectious Diseases. 2018;18(1):1-11.

117. Mann LM, Kirkcaldy RD, Papp JR, Torrone EA. Susceptibility of Neisseria gonorrhoeae to gentamicin—gonococcal isolate surveillance project, 2015–2016. Sexually transmitted diseases. 2018;45(2):96.

118. Harris SR, Cole MJ, Spiteri G, Sánchez-Busó L, Golparian D, Jacobsson S, et al. Public health surveillance of multidrug-resistant clones of Neisseria gonorrhoeae in Europe: a genomic survey. The Lancet Infectious Diseases. 2018;18(7):758-68.

119. Tayimetha CY, Unemo M. Antimicrobial susceptibility of Neisseria gonorrhoeae isolates in Yaoundé, Cameroon from 2009 to 2014. Sexually transmitted diseases. 2018;45(12):e101-e3.

120. Melendez JH, Hardick J, Barnes M, Page KR, Gaydos CA. Antimicrobial susceptibility of Neisseria gonorrhoeae isolates in Baltimore, Maryland, 2016: the importance of sentinel surveillance in the era of multi-drug-resistant gonorrhea. Antibiotics. 2018;7(3):77.

121. Liu Y-H, Huang Y-T, Liao C-H, Hsueh P-R. Antimicrobial susceptibilities and molecular typing of Neisseria gonorrhoeae isolates at a medical centre in Taiwan, 2001–2013 with an emphasis on high rate of azithromycin resistance among the isolates. International Journal of Antimicrobial Agents. 2018;51(5):768-74.

122. Kularatne R, Maseko V, Gumede L, Kufa T. Trends in Neisseria gonorrhoeae antimicrobial resistance over a ten-year surveillance period, Johannesburg, South Africa, 2008–2017. Antibiotics. 2018;7(3):58.

123. Phouangsouvanh S, Mayxay M, Keoluangkhot V, Vongsouvath M, Davong V, Dance DA. Antimicrobial susceptibility of Neisseria gonorrhoeae isolates in Vientiane, Lao PDR. Journal of global antimicrobial resistance. 2018;13:91-3.

124. Tshokey T, Tshering T, Pradhan AR, Adhikari D, Sharma R, Gurung K, et al. Antibiotic resistance in Neisseria gonorrhoea and treatment outcomes of gonococcal urethritis suspected patients in two large hospitals in Bhutan, 2015. PLoS One. 2018;13(8):e0201721.

125. Thakur SD, Levett PN, Horsman GB, Dillon J-AR. High levels of susceptibility to new and older antibiotics in Neisseria gonorrhoeae isolates from Saskatchewan (2003–15): time to consider point-of-care or molecular testing for precision treatment? Journal of Antimicrobial Chemotherapy. 2018;73(1):118-25.

126. Sirivongrangson P, Girdthep N, Sukwicha W, Buasakul P, Tongtoyai J, Weston E, et al. The first year of the global enhanced gonococcal antimicrobial surveillance programme (EGASP) in Bangkok, Thailand, 2015-2016. PloS one. 2018;13(11):e0206419.

127. Naznin M, Salam MA, Hossain MZ, Alam MS. Current status of gonococcal antimicrobial susceptibility with special reference to Azithromycin and Ceftriaxone: Report from a tertiary care hospital in Bangladesh. Pakistan Journal of Medical Sciences. 2018;34(6):1397.

128. Lee RS, Seemann T, Heffernan H, Kwong JC, Gonçalves da Silva A, Carter GP, et al. Genomic epidemiology and antimicrobial resistance of Neisseria gonorrhoeae in New Zealand. Journal of Antimicrobial Chemotherapy. 2018;73(2):353-64.

129. Kulkarni SV, Bala M, Muqeeth SA, Sasikala G, Nirmalkar AP, Thorat R, et al. Antibiotic susceptibility pattern of Neisseria gonorrhoeae strains isolated from five cities in India during 2013–2016. Journal of medical microbiology. 2018;67(1):22-8.

130. Jean S-S, Lu M-C, Shi Z-Y, Tseng S-H, Wu T-S, Lu P-L, et al. In vitro activity of ceftazidime–avibactam, ceftolozane–tazobactam, and other comparable agents against clinically important Gram-negative bacilli: Results from the 2017 Surveillance of Multicenter Antimicrobial Resistance in Taiwan (SMART). Infection and Drug Resistance. 2018;11:1983.

131. Gratrix J, Kamruzzaman A, Martin I, Smyczek P, Read R, Bertholet L, et al. Surveillance for antimicrobial resistance in gonorrhea: the Alberta model, 2012–2016. Antibiotics. 2018;7(3):63.

132. da Costa-Lourenço APR, Abrams AJ, Dos Santos KTB, Argentino ICV, Coelho-Souza T, Caniné MCA, et al. Phylogeny and antimicrobial resistance in Neisseria gonorrhoeae isolates from Rio de Janeiro, Brazil. Infection, Genetics and Evolution. 2018;58:157-63.

133. Bazzo M, Golfetto L, Gaspar P, Pires A, Ramos M, Franchini M, et al. First nationwide antimicrobial susceptibility surveillance for Neisseria gonorrhoeae in Brazil, 2015–16. Journal of Antimicrobial Chemotherapy. 2018;73(7):1854-61.

134. Yeshanew AG, Geremew RA. Neisseria Gonorrhoae and their antimicrobial susceptibility patterns among symptomatic patients from Gondar town, north West Ethiopia. Antimicrobial Resistance & Infection Control. 2018;7(1):1-7.

135. Calado J, Castro R, Lopes Â, Campos MJ, Rocha M, Pereira F. Antimicrobial resistance and molecular characteristics of Neisseria gonorrhoeae isolates from men who have sex with men. International Journal of Infectious Diseases. 2019;79:116-22.

136. Carannante A, Ciammaruconi A, Vacca P, Anselmo A, Fillo S, Palozzi AM, et al. Genomic characterization of gonococci from different anatomic sites, Italy, 2007–2014. Microbial Drug Resistance. 2019;25(9):1316-24.

137. Cobo F, Cabezas-Fernández MT, Avivar C. Typing and antimicrobial susceptibility of 134 Neisseria gonorrhoeae strains from Southern Spain. Revista Española de Quimioterapia. 2019;32(2):114.

138. Rambaran S, Naidoo K, Dookie N, Moodley P, Sturm AW. Resistance profile of Neisseria gonorrhoeae in KwaZulu-Natal, South Africa questioning the effect of the currently advocated dual therapy. Sexually transmitted diseases. 2019;46(4):266-70.

139. Kivata MW, Mbuchi M, Eyase FL, Bulimo WD, Kyanya CK, Oundo V, et al. gyrA and parC mutations in fluoroquinolone-resistant Neisseria gonorrhoeae isolates from Kenya. BMC microbiology. 2019;19(1):1-9.

140. Guerrero-Torres M, Menéndez M, Guerras C, Tello E, Ballesteros J, Clavo P, et al. Epidemiology, molecular characterisation and antimicrobial susceptibility of Neisseria gonorrhoeae isolates in Madrid, Spain, in 2016. Epidemiology & Infection. 2019;147.

141. Boiko I, Golparian D, Krynytska I, Bezkorovaina H, Frankenberg A, Onuchyna M, et al. Antimicrobial susceptibility of Neisseria gonorrhoeae isolates and treatment of gonorrhoea patients in Ternopil and Dnipropetrovsk regions of Ukraine, 2013–2018. Apmis. 2019;127(7):503-9.

142. Yang Y, Yang Y, Martin I, Dong Y, Diao N, Wang Y, et al. NG-STAR genotypes are associated with MDR in Neisseria gonorrhoeae isolates collected in 2017 in Shanghai. Journal of Antimicrobial Chemotherapy. 2020;75(3):566-70.

143. Yéo A, Kouamé-Blavo B, Kouamé CE, Ouattara A, Yao AC, Gbedé BD, et al. Establishment of a Gonococcal Antimicrobial Surveillance Programme, in Accordance With World Health Organization Standards, in Côte d'Ivoire, Western Africa, 2014–2017. Sexually Transmitted Diseases. 2019;46(3):179-84.

144. Martins RA, Cassu-Corsi D, Nodari CS, Cayô R, Natsumeda L, Streling AP, et al. Temporal evolution of antimicrobial resistance among Neisseria gonorrhoeae clinical isolates in the most populated South American Metropolitan Region. Memórias do Instituto Oswaldo Cruz. 2019;114.

145. Liu J-W, Xu W-Q, Zhu X-Y, Dai X-Q, Chen S-C, Han Y, et al. Gentamicin susceptibility of Neisseria gonorrhoeae isolates from 7 provinces in China. Infection and Drug Resistance. 2019;12:2471.

146. Gianecini RA, Golparian D, Zittermann S, Litvik A, Gonzalez S, Oviedo C, et al. Genome-based epidemiology and antimicrobial resistance determinants of Neisseria gonorrhoeae isolates with decreased susceptibility and resistance to extended-spectrum cephalosporins in Argentina in 2011–16. Journal of Antimicrobial Chemotherapy. 2019;74(6):1551-9.

147. Bailey AL, Potter RF, Wallace MA, Johnson C, Dantas G, Burnham C-AD. Genotypic and phenotypic characterization of antimicrobial resistance in Neisseria gonorrhoeae: a cross-sectional study of isolates recovered from routine urine cultures in a high-incidence setting. MSphere. 2019;4(4):e00373-19.

148. Visser M, van Westreenen M, van Bergen J, van Benthem BH. Low gonorrhoea antimicrobial resistance and culture positivity rates in general practice: a pilot study. Sexually transmitted infections. 2020;96(3):220-2.

149. Kubanov A, Solomka V, Plakhova X, Chestkov A, Petrova N, Shaskolskiy B, et al. Summary and trends of the Russian gonococcal antimicrobial surveillance programme, 2005 to 2016. Journal of clinical microbiology. 2019;57(6):e02024-18.

150. Indriatmi W, Prayogo RL, Nilasari H, Suseno LS. Antimicrobial resistance of Neisseria gonorrhoeae in Jakarta, Indonesia: a cross-sectional study. Sexual Health. 2019;17(1):9-14.

151. Yan J, Xue J, Chen Y, Chen S, Wang Q, Zhang C, et al. Increasing prevalence of Neisseria gonorrhoeae with decreased susceptibility to ceftriaxone and resistance to azithromycin in Hangzhou, China (2015–17). Journal of Antimicrobial Chemotherapy. 2019;74(1):29-37.

152. Qin X, Zhao Y, Chen W, Wu X, Tang S, Li G, et al. Changing antimicrobial susceptibility and molecular characterisation of Neisseria gonorrhoeae isolates in Guangdong, China: in a background of rapidly rising epidemic. International journal of antimicrobial agents. 2019;54(6):757-65.

153. Mabonga E, Parkes-Ratanshi R, Riedel S, Nabweyambo S, Mbabazi O, Taylor C, et al. Complete ciprofloxacin resistance in gonococcal isolates in an urban Ugandan clinic: findings from a cross-sectional study. International journal of STD & AIDS. 2019;30(3):256-63.

154. Martin I, Sawatzky P, Allen V, Lefebvre B, Hoang L, Naidu P, et al. Multidrug resistant gonorrhea: Multidrug-resistant and extensively drug-resistant Neisseria gonorrhoeae in Canada, 2012–2016. Canada Communicable Disease Report. 2019;45(2-3):45.

155. Attram N, Agbodzi B, Dela H, Behene E, Nyarko EO, Kyei NN, et al. Antimicrobial resistance (AMR) and molecular characterization of Neisseria gonorrhoeae in Ghana, 2012-2015. PloS one. 2019;14(10):e0223598.

156. Golparian D, Harris SR, Sánchez-Busó L, Hoffmann S, Shafer WM, Bentley SD, et al. Genomic evolution of Neisseria gonorrhoeae since the preantibiotic era (1928–2013): antimicrobial use/misuse selects for resistance and drives evolution. BMC genomics. 2020;21(1):1-13.

157. Queirós C, da Costa JB, Lito L, Filipe P, Cristino JM. Estudio retrospectivo acerca de la evolución y el desarrollo de resistencias antimicrobianas en casos diagnosticados de gonorrea en un hospital de nivel terciario en Portugal durante 10 años. Actas Dermo-Sifiliográficas. 2020;111(9):761-7.

158. Lan PT, Golparian D, Ringlander J, Van Hung L, Van Thuong N, Unemo M. Genomic analysis and antimicrobial resistance of Neisseria gonorrhoeae isolates from Vietnam in 2011 and 2015–16. Journal of Antimicrobial Chemotherapy. 2020;75(6):1432-8.

159. Pinto M, Rodrigues JC, Matias R, Água-Doce I, Cordeiro D, Correia C, et al. Fifteen years of a nationwide culture collection of Neisseria gonorrhoeae antimicrobial resistance in Portugal. European Journal of Clinical Microbiology & Infectious Diseases. 2020;39(9):1761-70.

160. Boiko I, Golparian D, Jacobsson S, Krynytska I, Frankenberg A, Shevchenko T, et al. Genomic epidemiology and antimicrobial resistance determinants of Neisseria gonorrhoeae isolates from Ukraine, 2013–2018. Apmis. 2020;128(7):465-75.

161. García UI, Toboso MCN, Azpeitia EM, Perez MI, Ragpa LH, Menica JAÁ, et al. Epidemiological surveillance study of gonococcal infection in Northern Spain. Enfermedades Infecciosas y Microbiología Clínica. 2020;38(2):59-64.

162. Alfsnes K, Eldholm V, Olsen AO, Brynildsrud OB, Bohlin J, Steinbakk M, et al. Genomic epidemiology and population structure of Neisseria gonorrhoeae in Norway, 2016–2017. Microbial genomics. 2020;6(4).

163. Town K, Harris S, Sánchez-Busó L, Cole MJ, Pitt R, Fifer H, et al. Genomic and phenotypic variability in Neisseria gonorrhoeae antimicrobial susceptibility, England. Emerging infectious diseases. 2020;26(3):505.

164. Maduna LD, Kock MM, Van der Veer BM, Radebe O, McIntyre J, Van Alphen LB, et al. Antimicrobial resistance of Neisseria gonorrhoeae isolates from high-risk men in Johannesburg, South Africa. Antimicrobial agents and chemotherapy. 2020;64(11):e00906-20.

165. Golparian D, Bazzo ML, Golfetto L, Gaspar PC, Schörner MA, Schwartz Benzaken A, et al. Genomic epidemiology of Neisseria gonorrhoeae elucidating the gonococcal antimicrobial resistance and lineages/sublineages across Brazil, 2015–16. Journal of Antimicrobial Chemotherapy. 2020;75(11):3163-72.

166. Dong Y, Yang Y, Wang Y, Martin I, Demczuk W, Gu W. Shanghai Neisseria gonorrhoeae isolates exhibit resistance to extended-spectrum cephalosporins and clonal distribution. Frontiers in microbiology. 2020;11:580399.

167. Crucitti T, Belinga S, Fonkoua M, Abanda M, Mbanzouen W, Sokeng E, et al. Sharp increase in ciprofloxacin resistance of Neisseria gonorrhoeae in Yaounde, Cameroon: analyses of a laboratory database period 2012–2018. International journal of STD & AIDS. 2020;31(6):579-86.

168. Salmerón P, Viñado B, El Ouazzani R, Hernández M, Barbera MJ, Alberny M, et al. Antimicrobial susceptibility of Neisseria gonorrhoeae in Barcelona during a five-year period, 2013 to 2017. Eurosurveillance. 2020;25(42):1900576.

169. Wang F, Liu J-W, Li Y-Z, Zhang L-J, Huang J, Chen X-S, et al. Surveillance and molecular epidemiology of Neisseria gonorrhoeae isolates in Shenzhen, China, 2010–2017. Journal of Global Antimicrobial Resistance. 2020;23:269-74.

170. Nacht C, Agingu W, Otieno F, Odhiambo F, Mehta SD. Antimicrobial resistance patterns in Neisseria gonorrhoeae among male clients of a sexually transmitted infections clinic in Kisumu, Kenya. International journal of STD & AIDS. 2020;31(1):46-52.

171. Mortimer TD, Pathela P, Crawley A, Rakeman JL, Lin Y, Harris SR, et al. The distribution and spread of susceptible and resistant Neisseria gonorrhoeae across demographic groups in a major metropolitan center. Clinical Infectious Diseases. 2021;73(9):e3146-e55.

172. Kularatne R, Kufa T, Gumede L, Maseko V. Comparison of gentamicin MICs by agar dilution and Etest for clinical isolates of Neisseria gonorrhoeae. Journal of Antimicrobial Chemotherapy. 2020;75(9):2599-604.

173. Fentaw S, Abubeker R, Asamene N, Assefa M, Bekele Y, Tigabu E. Antimicrobial susceptibility profile of Gonococcal isolates obtained from men presenting with urethral discharge in Addis Ababa, Ethiopia: Implications for national syndromic treatment guideline. PloS one. 2020;15(6):e0233753.

174. Adamson PC, Van Le H, Le HHL, Le GM, Nguyen TV, Klausner JD. Trends in antimicrobial resistance in Neisseria gonorrhoeae in Hanoi, Vietnam, 2017–2019. BMC infectious diseases. 2020;20(1):1-6.

175. Zheng Z, Liu L, Shen X, Yu J, Chen L, Zhan L, et al. Antimicrobial resistance and molecular characteristics among Neisseria gonorrhoeae clinical isolates in a Chinese tertiary hospital. Infection and Drug Resistance. 2019;12:3301.

176. Sahile A, Teshager L, Fekadie M, Gashaw M. Prevalence and Antimicrobial Susceptibility Patterns of Neisseria gonorrhoeae among Suspected Patients Attending Private Clinics in Jimma, Ethiopia. International Journal of Microbiology. 2020;2020.

177. Unemo M, Ahlstrand J, Sánchez-Busó L, Day M, Aanensen D, Golparian D, et al. High susceptibility to zoliflodacin and conserved target (GyrB) for zoliflodacin among 1209 consecutive clinical Neisseria gonorrhoeae isolates from 25 European countries, 2018. Journal of Antimicrobial Chemotherapy. 2021;76(5):1221-8.

178. Yan J, Chen Y, Yang F, Ling X, Jiang S, Zhao F, et al. High percentage of the ceftriaxone-resistant Neisseria gonorrhoeae FC428 clone among isolates from a single hospital in Hangzhou, China. Journal of Antimicrobial Chemotherapy. 2021;76(4):936-9.

179. Tanaka M, Furuya R, Kobayashi I, Ohno A, Kanesaka I. Molecular characteristics and antimicrobial susceptibility of penicillinase-producing Neisseria gonorrhoeae isolates in Fukuoka, Japan, 1996–2018. Journal of Global Antimicrobial Resistance. 2021;26:45-51.

180. Jamoralin Jr MC, Argimón S, Lagrada ML, Villamin AS, Masim ML, Gayeta JM, et al. Genomic surveillance of Neisseria gonorrhoeae in the Philippines, 2013–2014. Western Pacific Surveillance and Response Journal: WPSAR. 2021;12(1):17.

181. de Korne-Elenbaas J, Bruisten SM, de Vries HJ, Van Dam AP. Emergence of a Neisseria gonorrhoeae clone with reduced cephalosporin susceptibility between 2014 and 2019 in Amsterdam, The Netherlands, revealed by genomic population analysis. Journal of Antimicrobial Chemotherapy. 2021;76(7):1759-68.

182. Aniskevich A, Shimanskaya I, Boiko I, Golubovskaya T, Golparian D, Stanislavova I, et al. Antimicrobial resistance in Neisseria gonorrhoeae isolates and gonorrhoea treatment in the Republic of Belarus, Eastern Europe, 2009–2019. BMC Infectious Diseases. 2021;21(1):1-9.

183. Salmerón P, Viñado B, Arando M, Alcoceba E, Romero B, Menéndez B, et al. Neisseria gonorrhoeae antimicrobial resistance in Spain: a prospective multicentre study. Journal of Antimicrobial Chemotherapy. 2021;76(6):1523-31.

184. Karymbaeva S, Boiko I, Jacobsson S, Mamaeva G, Ibraeva A, Usupova D, et al. Antimicrobial resistance and molecular epidemiological typing of Neisseria gonorrhoeae isolates from Kyrgyzstan in Central Asia, 2012 and 2017. BMC Infectious Diseases. 2021;21(1):1-9.

185. Kakooza F, Musinguzi P, Workneh M, Walwema R, Kyambadde P, Mande E, et al. Implementation of a standardised and quality-assured enhanced gonococcal antimicrobial surveillance programme in accordance with WHO protocols in Kampala, Uganda. Sexually transmitted infections. 2021;97(4):312-6.

186. Jacobsson S, Cole MJ, Spiteri G, Day M, Unemo M. Associations between antimicrobial susceptibility/resistance of Neisseria gonorrhoeae isolates in European Union/European Economic Area and patients’ gender, sexual orientation and anatomical site of infection, 2009–2016. BMC infectious diseases. 2021;21(1):1-12.

187. Zhu B, Hu Y, Zhou X, Liu K, Wen W, Hu Y. Retrospective Analysis of Drug Sensitivity of Neisseria gonorrhoeae in Teaching Hospitals of South China. Infection and Drug Resistance. 2021;14:2087.

188. Le W, Su X, Lou X, Li X, Gong X, Wang B, et al. Susceptibility Trends of Zoliflodacin against Multidrug-Resistant Neisseria gonorrhoeae Clinical Isolates in Nanjing, China, 2014 to 2018. Antimicrobial Agents and Chemotherapy. 2021;65(3):e00863-20.

**Systematic literature search**

**Screening and eligibility of records**

Our initial search yielded 2350 reports. After removing 125 duplicates, 2225 unique reports remained. Following title and abstract screening, 1945 reports were excluded based on the following reasons:

Not relevant to the research question: 1,015

Not meeting study design criteria: 620

Incorrect population: 210

Outcome not measured: 100
