# Supplementary material for: Global trends of antimicrobial resistance rates in Neisseria gonorrhoeae: a systematic review and meta-analysis
Source: Front Pharmacol. 2024 Jul 3;15:1284665. doi: 10.3389/fphar.2024.1284665 (PMC11258497; doi:10.3389/fphar.2024.1284665)

**Supplementary Figure S2.**


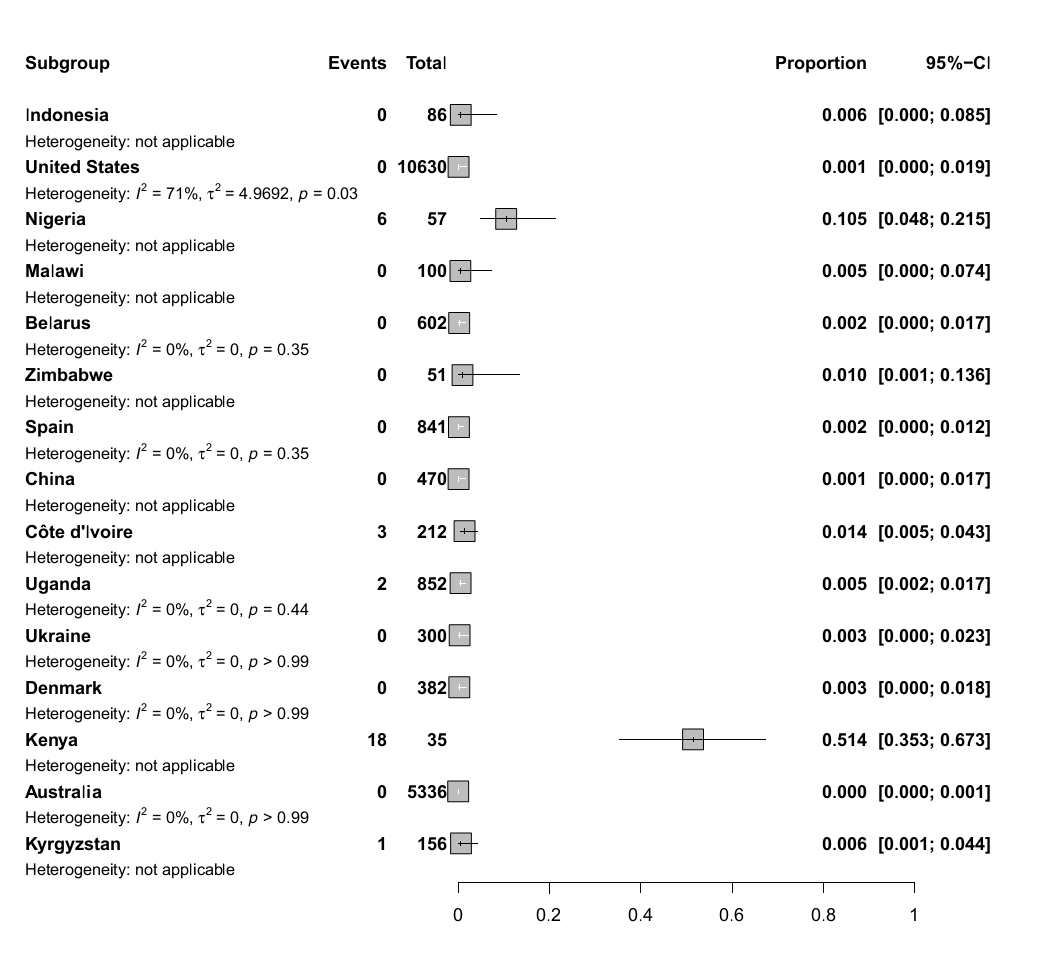


Forest plot for the gentamicin resistance rate stratified by countries.


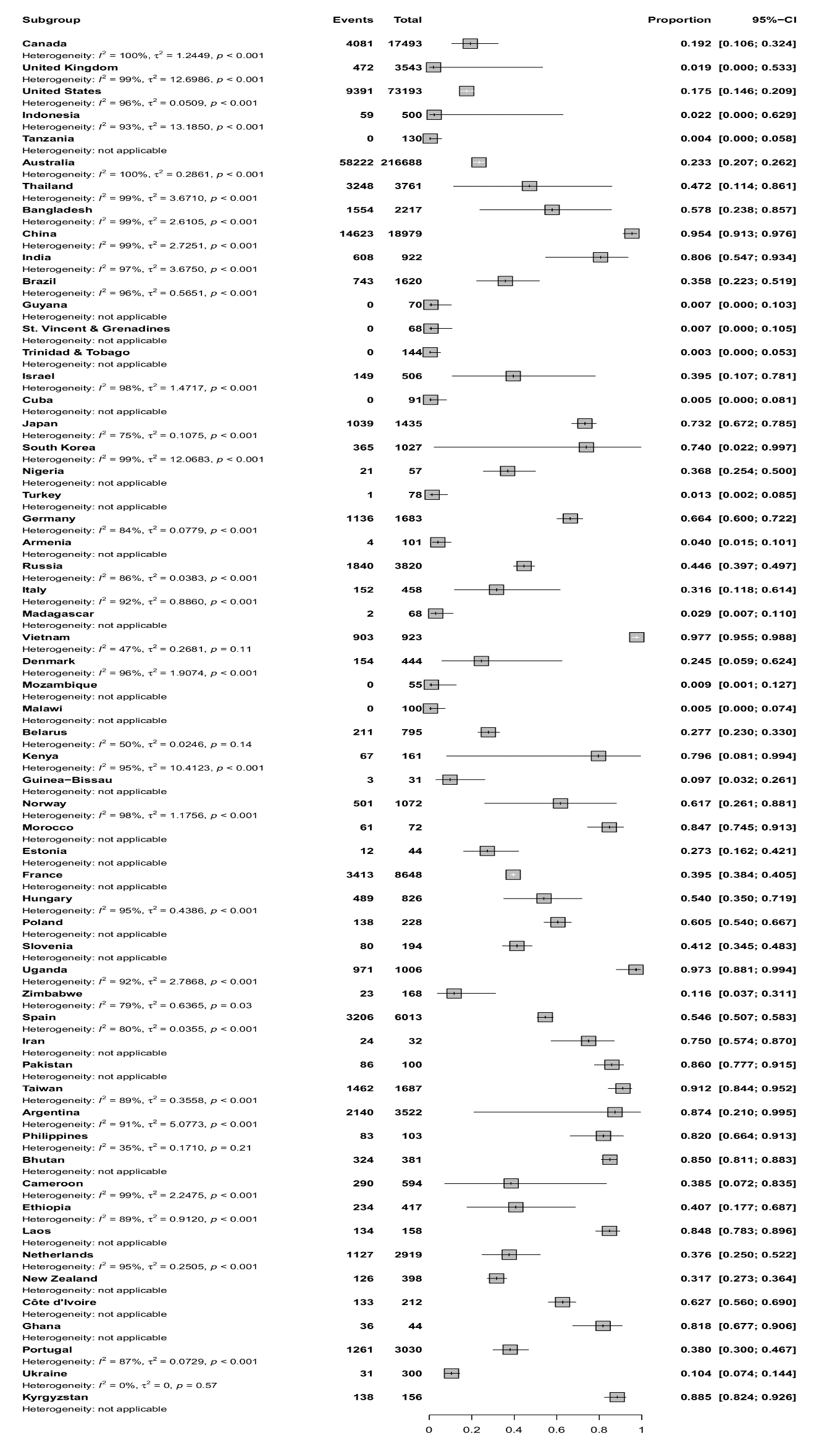


Forest plot for the ciprofloxacin resistance rate stratified by countries.


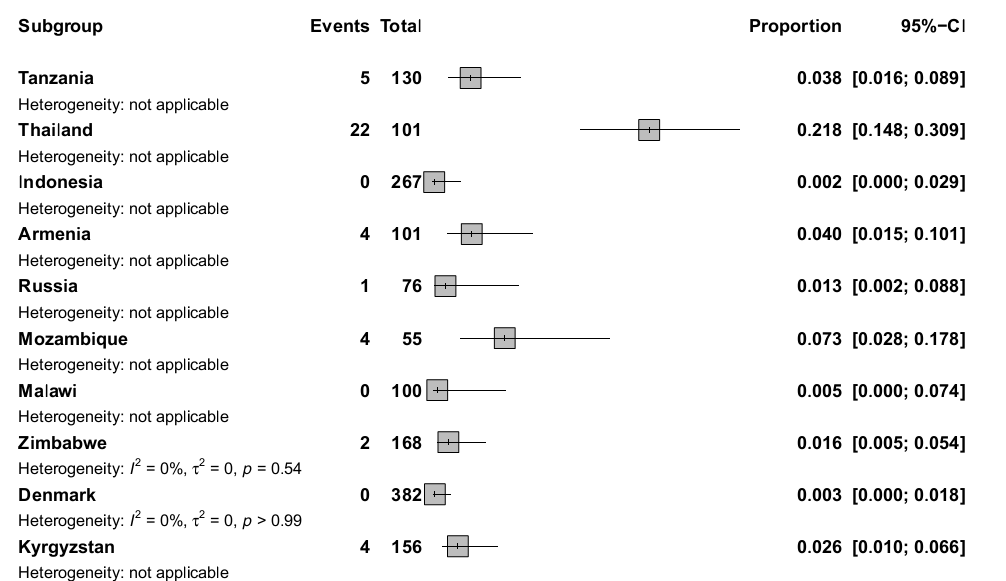


Forest plot for the kanamycin resistance rate stratified by countries.


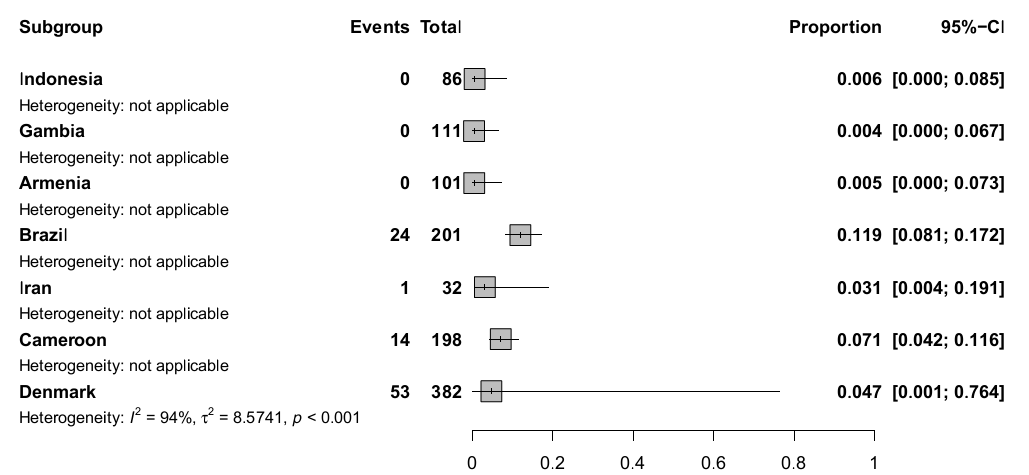


Forest plot for the chloramphenicol resistance rate stratified by countries.


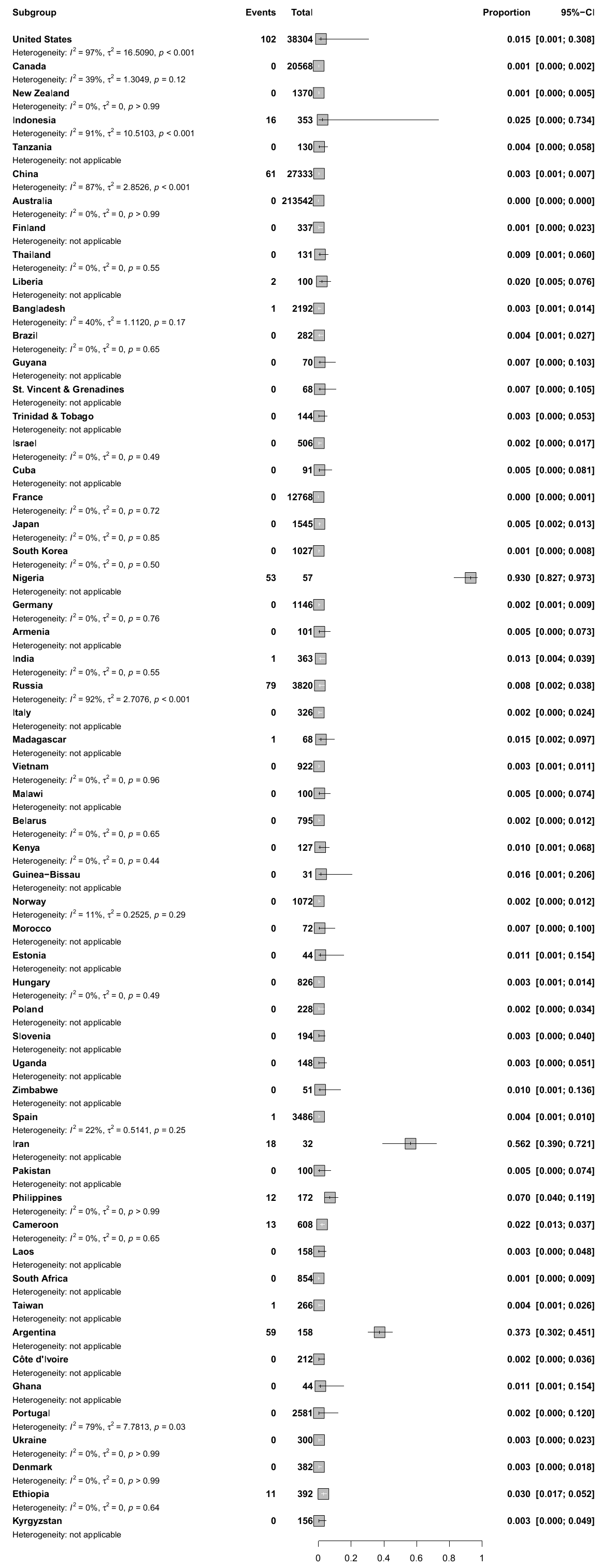


Forest plot for the spectinomycin resistance rate stratified by countries.


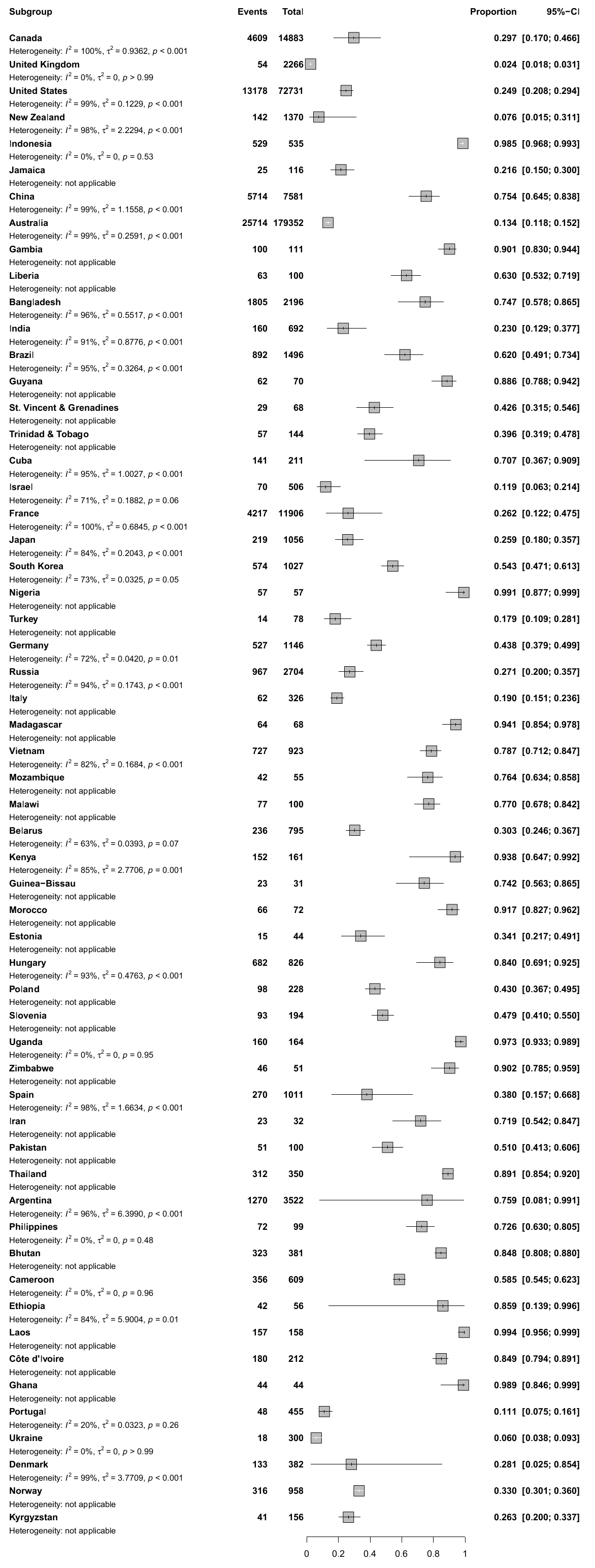


Forest plot for the tetracycline resistance rate stratified by countries.

- **Funnel plots depicts publication bias of included studies on antibiotic resistance**

**
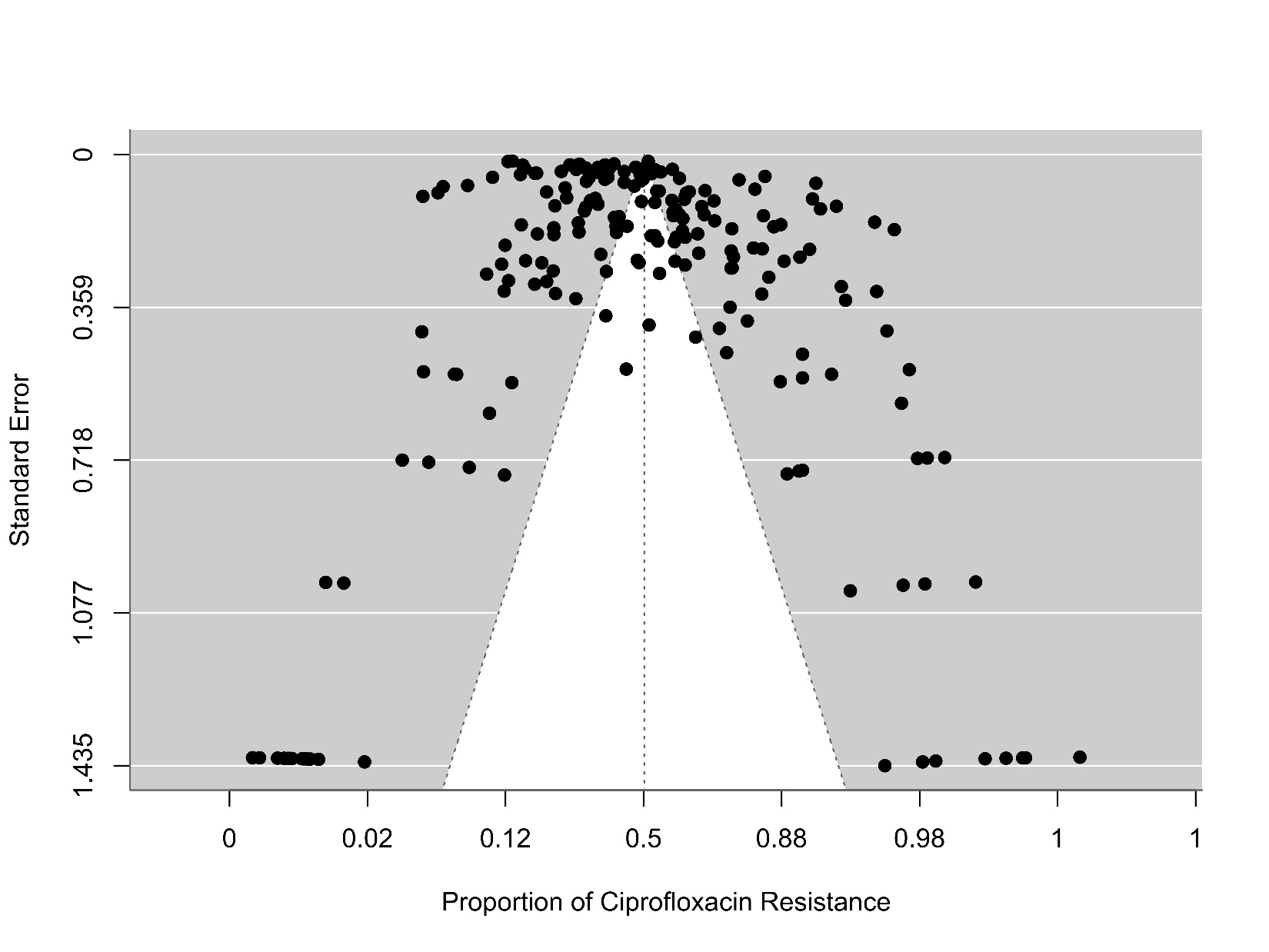
**


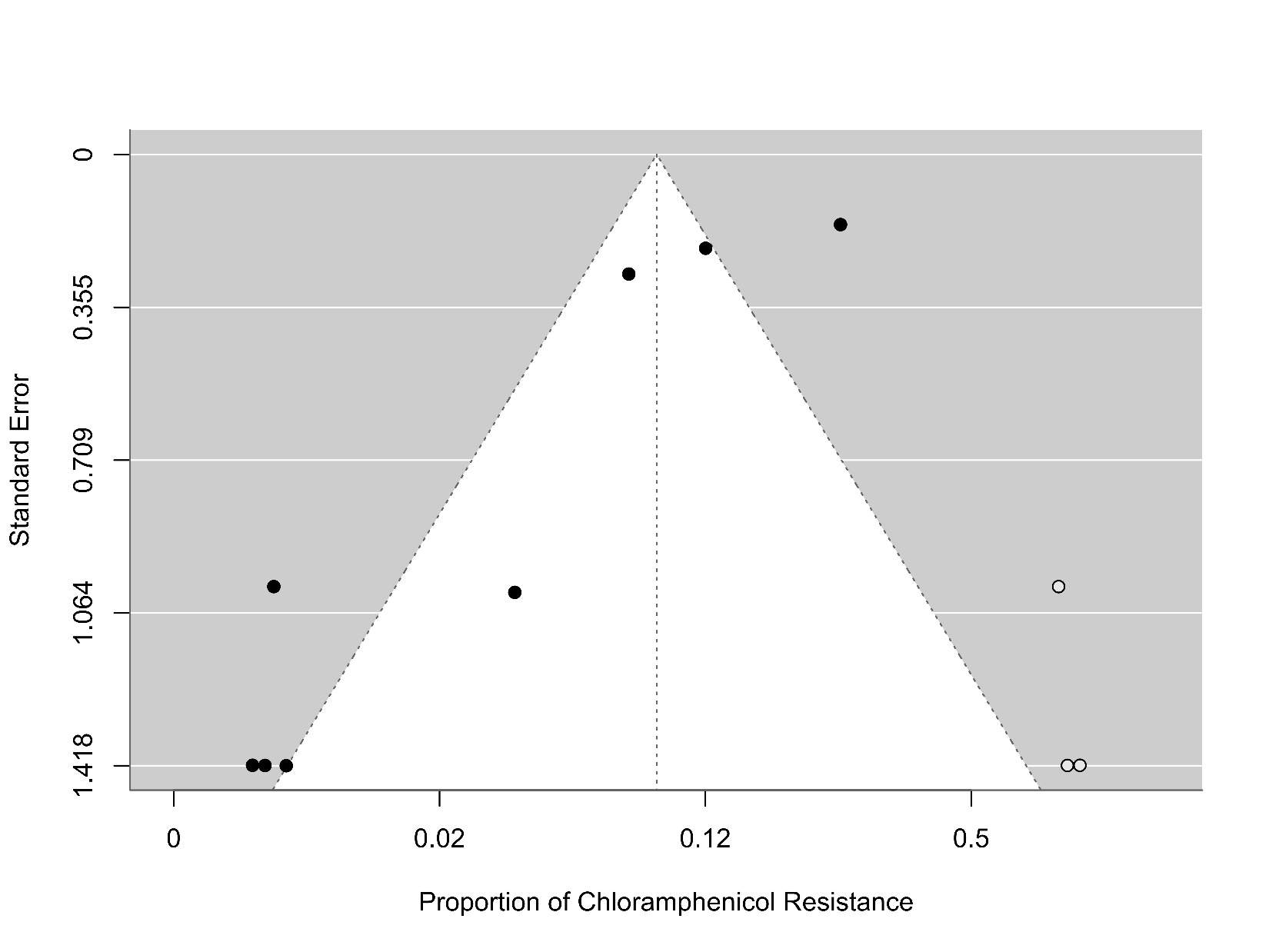


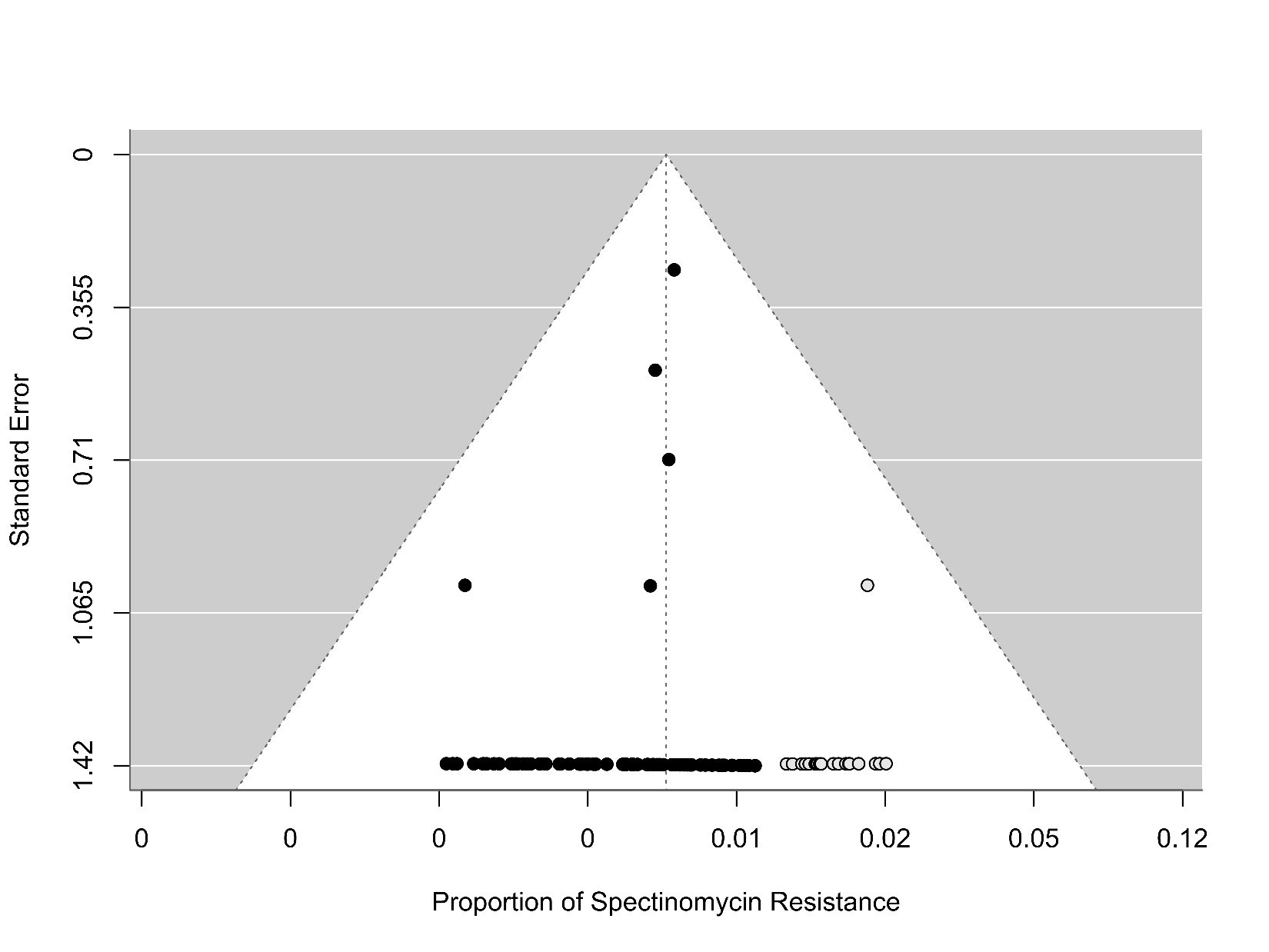

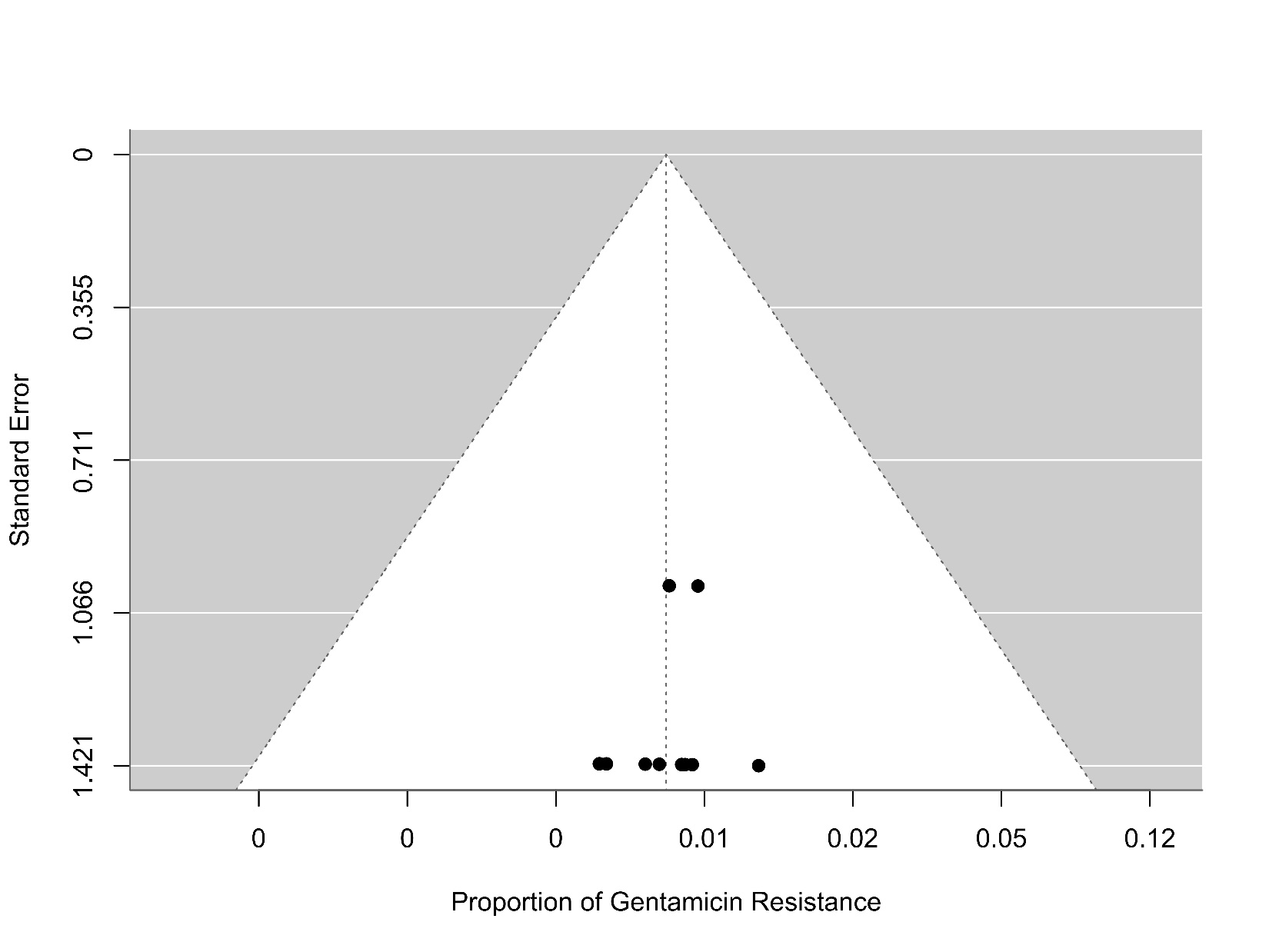


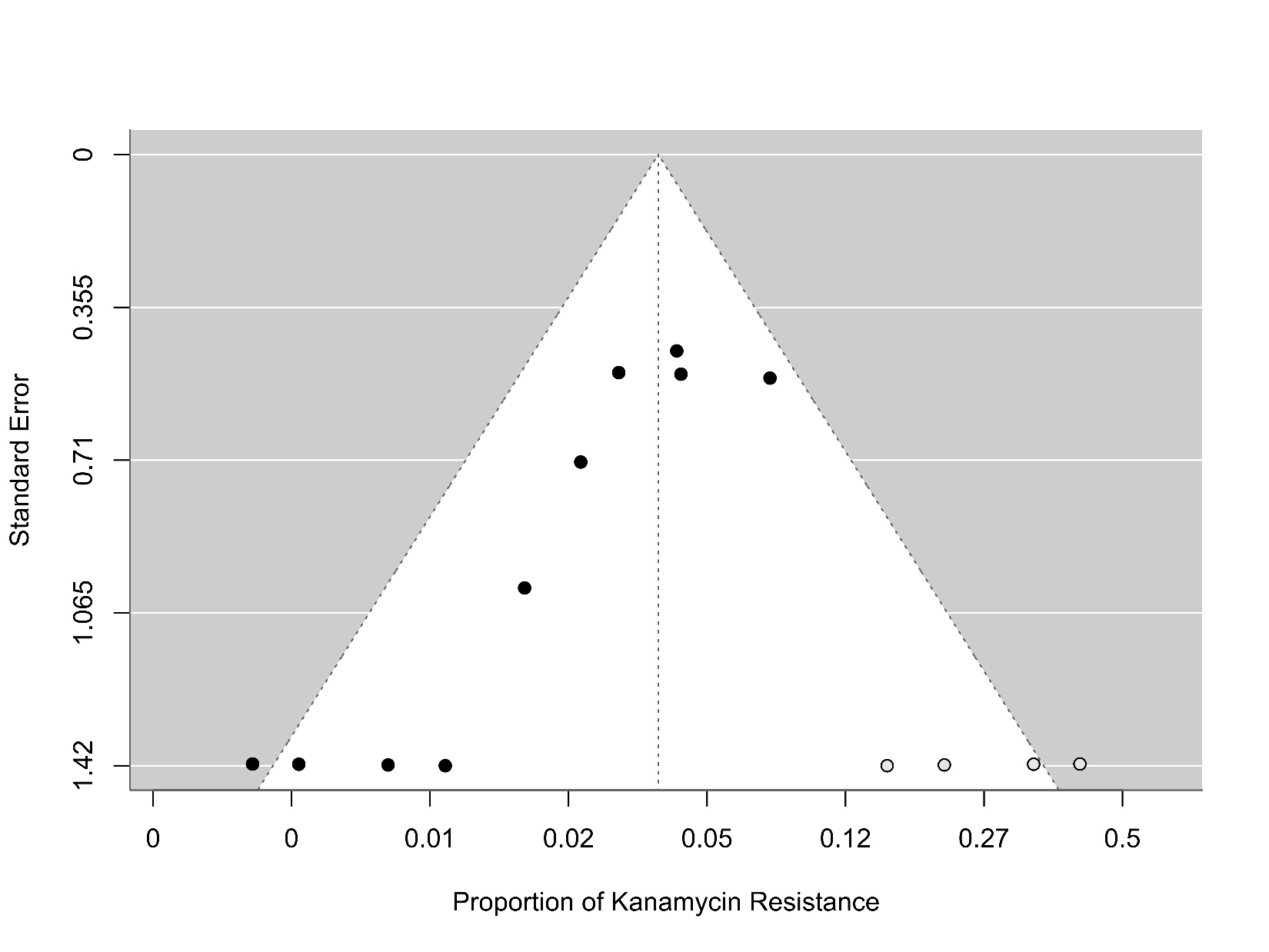


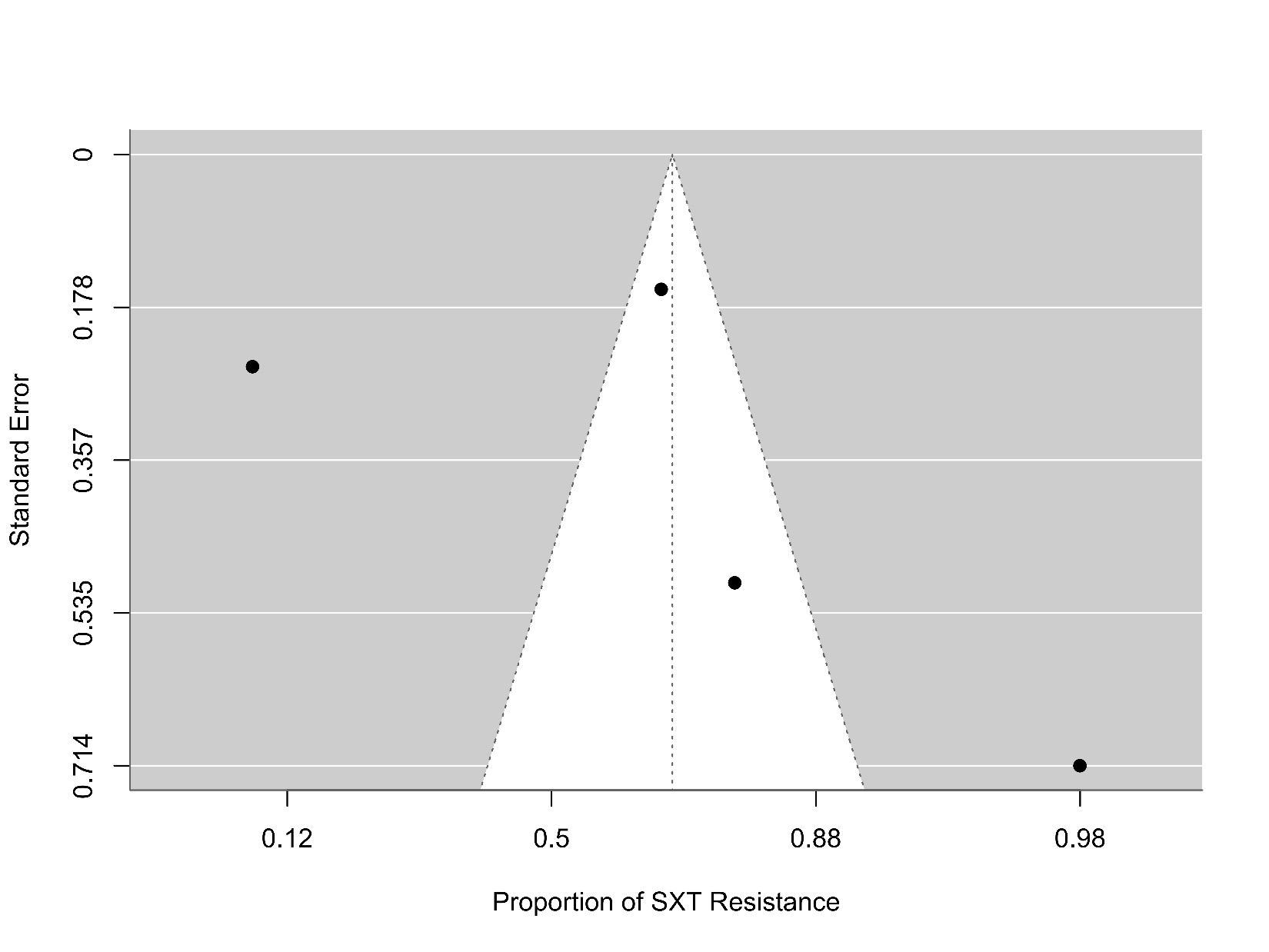

Supplement: Supplementary file 2 [file DataSheet2.docx]
