# Supplementary material for: Global trends of antimicrobial resistance rates in Neisseria gonorrhoeae: a systematic review and meta-analysis
Source: Front Pharmacol. 2024 Jul 3;15:1284665. doi: 10.3389/fphar.2024.1284665 (PMC11258497; doi:10.3389/fphar.2024.1284665)

**
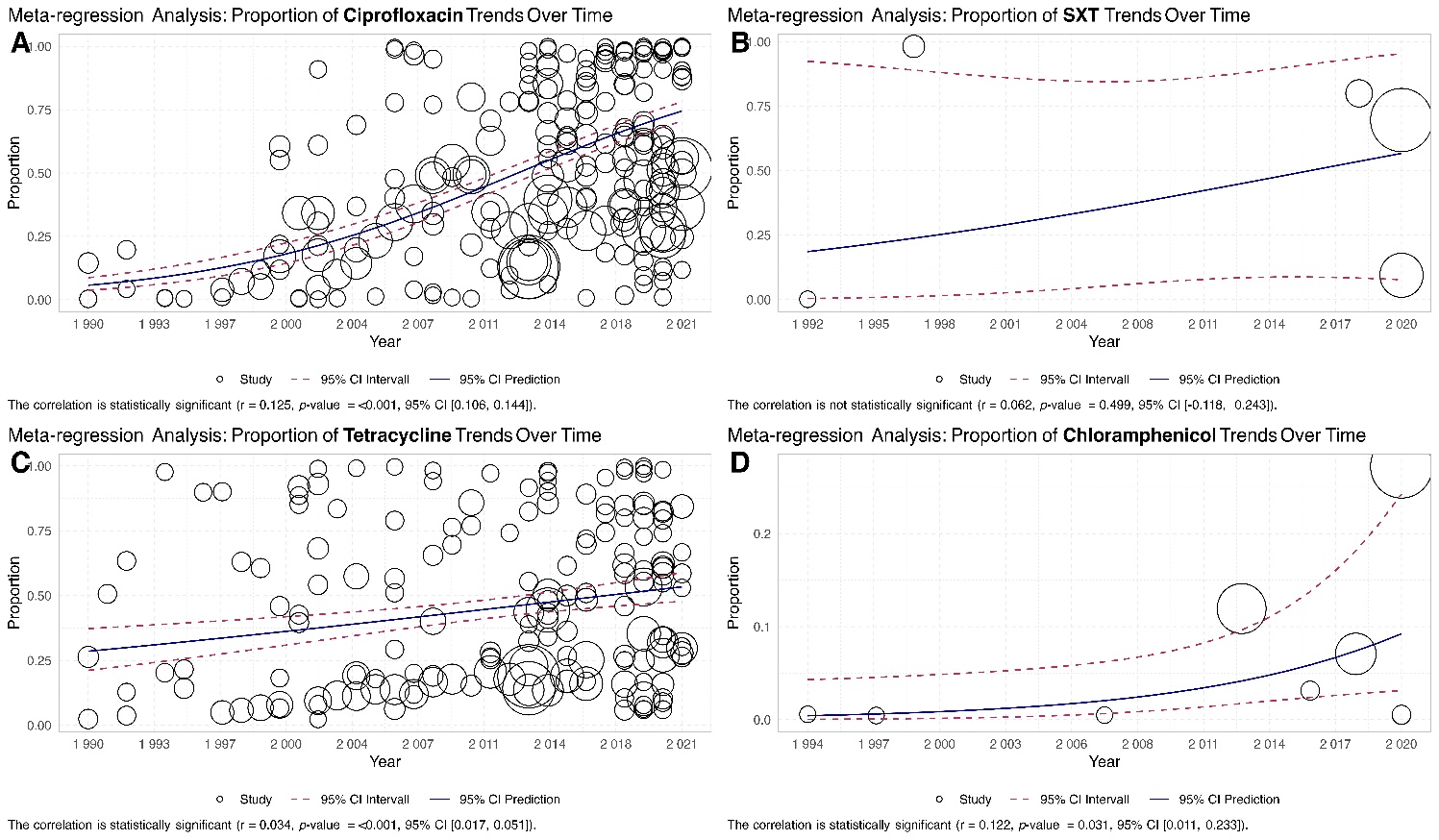
**

**Figure. Meta-regression results for changing antibiotic resistance rate over time.**

Regression of rates of Ciprofloxacin, Tetracycline, Spectinomycin resistance in China, Australia, and USA over time.

# China

### Ciprofloxacin


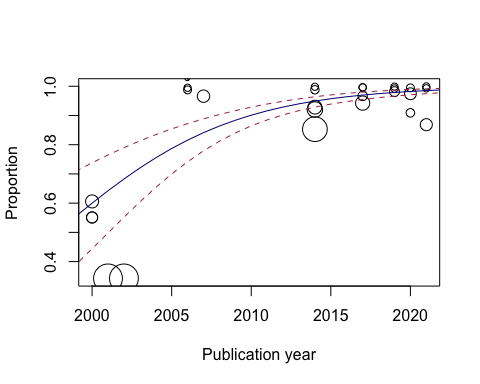


###

# China

### Tetracycline


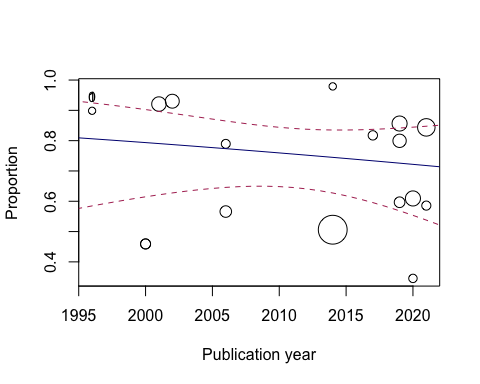


# China

### Spectinomycin


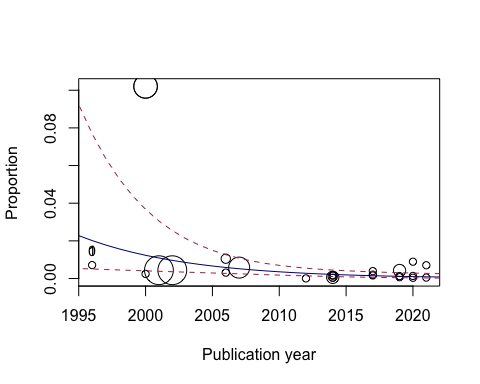


# Australia

### Ciprofloxacin


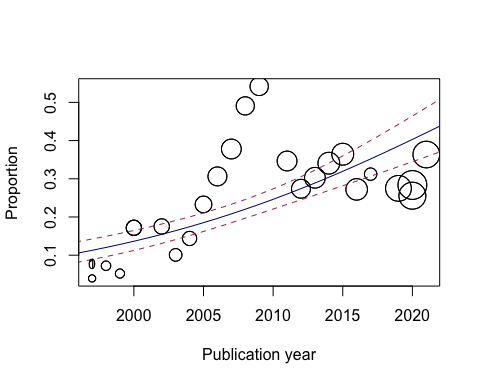


# Australia

### Tetracycline


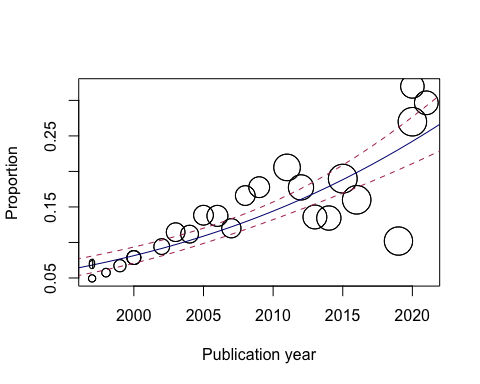


# Australia

### Spectinomycin


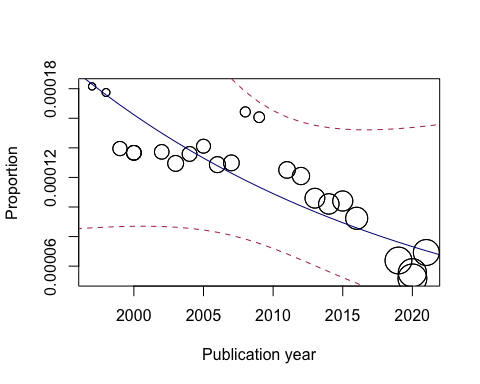


# USA

### Ciprofloxacin


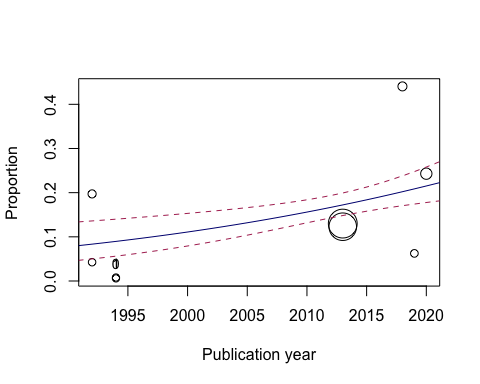


# USA

### Tetracycline


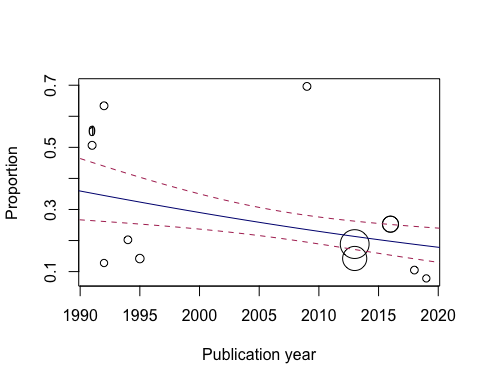


# USA

**Spectinomycin**


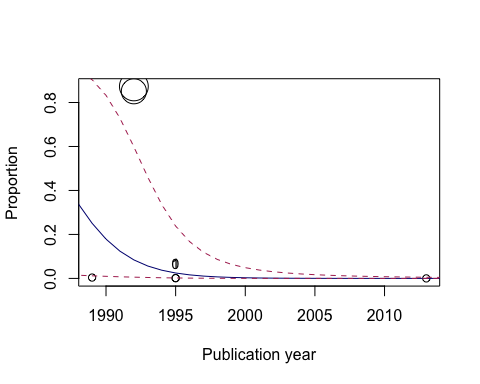

Supplement: Supplementary file 3 [file DataSheet1.docx]
